# Extensive sequence variation in rice blast resistance gene *Pi54* makes it broad spectrum in nature

**DOI:** 10.3389/fpls.2015.00345

**Published:** 2015-05-21

**Authors:** Shallu Thakur, Pankaj K. Singh, Alok Das, R. Rathour, M. Variar, S. K. Prashanthi, A. K. Singh, U. D. Singh, Duni Chand, N. K. Singh, Tilak R. Sharma

**Affiliations:** ^1^National Research Centre on Plant Biotechnology, Pusa CampusNew Delhi, India; ^2^Department of Biotechnology, Himachal Pradesh UniversityShimla, India; ^3^Department of Agricultural Biotechnology, CSK Himachal Pradesh Agricultural UniversityPalampur, India; ^4^Central Rainfed Upland Rice Research Station, Central Rice Research InstituteHazaribagh, India; ^5^School of Agricultural Biotechnology, University of Agricultural SciencesDharwad, India; ^6^Indian Agricultural Research InstituteNew Delhi, India

**Keywords:** allele mining, *Pi54*, rice landraces, polymorphism, blast resistance genes, allele specific markers

## Abstract

Rice blast resistant gene, *Pi54* cloned from rice line, Tetep, is effective against diverse isolates of *Magnaporthe oryzae*. In this study, we prospected the allelic variants of the dominant blast resistance gene from a set of 92 rice lines to determine the nucleotide diversity, pattern of its molecular evolution, phylogenetic relationships and evolutionary dynamics, and to develop allele specific markers. High quality sequences were generated for homologs of *Pi54* gene. Using comparative sequence analysis, InDels of variable sizes in all the alleles were observed. Profiling of the selected sites of SNP (Single Nucleotide Polymorphism) and amino acids (N sites ≥ 10) exhibited constant frequency distribution of mutational and substitutional sites between the resistance and susceptible rice lines, respectively. A total of 50 new haplotypes based on the nucleotide polymorphism was also identified. A unique haplotype (H_3) was found to be linked to all the resistant alleles isolated from *indica* rice lines. Unique leucine zipper and tyrosine sulfation sites were identified in the predicted Pi54 proteins. Selection signals were observed in entire coding sequence of resistance alleles, as compared to LRR domains for susceptible alleles. This is a maiden report of extensive variability of *Pi54* alleles in different landraces and cultivated varieties, possibly, attributing broad-spectrum resistance to *Magnaporthe oryzae*. The sequence variation in two consensus region: 163 and 144 bp were used for the development of allele specific DNA markers. Validated markers can be used for the selection and identification of better allele(s) and their introgression in commercial rice cultivars employing marker assisted selection.

## Introduction

Blast disease caused by the fungus, *Magnaporthe oryzae* is one of the most widespread and devastating diseases of rice. Management of rice blast through host resistance is a promising component of the Integrated Disease Management (IDM) programme. Till date, about 101 major rice blast resistance (*R*) genes have been identified, and 20 of them cloned and characterized (Sharma et al., 2012). Numerous R-genes identified, cloned and characterized are categorized in eight classes based on their amino acid motif organization (Sharma et al., 2014). Majority of loci associated with rice blast disease resistance have been reported on chromosome 11 of rice based on genome wide association studies (Wang et al., 2014). Although several blast resistance loci have been identified but only few of them has been employed in breeding for blast management in India (Singh et al., 2011). Further limited success has been realized in durable resistance breeding programmes due to variability of pathogen across locations. Harnessing rice diversity adapted in farmers' fields over the years appears promising alternative to look for resistance source.

Exploring the genetic variants from germplasm (wild and cultivated) is currently being envisaged in many crop species. One of the most widely used methods to identify variants employs polymerase chain reaction (PCR) based techniques to amplify homologs (possible alleles) from the gene pool, known as *allele mining*. Recently, allele mining for blast resistance has been reported from wild and cultivated species of rice (Yang et al., 2007; Geng et al., 2008; Huang et al., 2008). Studies of *Pi-ta* gene in rice lines including wild (AA and CC genome) and cultivated species indicated consensus conserved sequence before divergence (Wang et al., 2008). In another study, *Pi-ta* orthologs from 26 accessions (*Oryza rufipogon, O. sativa, O. meridionalis*, and *O. officinalis*), collected from 10 different countries highlighted dimorphic pattern of nucleotide polymorphism and low nucleotide diversity at the LRD region (Yoshida and Miyashita, 2009). In similar lines, the allelic variants and flanking sequences of *Pi-ta* have been studied in 159 geographically diverse accessions of *Oryza* species (AA genome) (Lee et al., 2009). The *Pi-ta* alleles also have been studied extensively in Indian landraces (Thakur et al., 2013b). Other blast resistance loci like *Pid3, Pi9 and Piz(t)* has been explored to study the nucleotide polymorphism and evolutionary pressure (Shang et al., 2009; Liu et al., 2011; Thakur et al., 2013a). However, such detailed analysis is lacking for the important blast resistance gene, *Pi54* that confers broad spectrum resistance to blast disease (Sharma et al., 2010). The *Pi54* gene located on chromosome 11 having unique zinc finger domain, besides LRR domain (Sharma et al., 2005a,b; Gupta et al., 2012). Functional complementation indicated that this gene provides stable and high level of resistance against geographically diverse strains of *M. oryzae*, collected from different parts of India (Rai et al., 2011). The gene possibly triggers up-regulation of defense response genes (callose, laccase, PAL, and peroxidase), transcription factors (NAC6, Dof Zinc finger, MAD box, bZIP, and WRKY) that fortify cell wall/plasmodesmata leading to hypersensitive response, and affecting resistance reaction (Gupta et al., 2011). Currently, the gene is being used in enhanced blast resistant breeding programme (Singh et al., 2011). An ortholog of *Pi54* gene from wild species of rice has also been recently cloned and functionally validated (Das et al., 2012). However, the allelic variants of *Pi54* gene have not been characterized from rice landraces, that are believed to have co-evolved with pathogen, and hence represents better “evo-devo” perspective of resistance reaction.

Till date, cultivated and wild species of rice have been employed for prospecting novel variants of blast resistance genes. Landraces too represents unmatched genetic potential for rice improvement. The local landraces or local rice varieties are genetically diverse, balanced population and are in equilibrium with the environment and pathogens, in contrast to the rice varieties. Unlike high yielding varieties, the landraces are endowed with tremendous genetic variability, as they are not subjected to subtle selection over a long period of time. Probably, it helps landraces to adapt in wide agro-ecological niches with unmatched qualitative traits, medicinal properties and important genetic resources for resistance to pests and diseases. Owing to their specific domination in geo-graphical niches, landraces have genes of resistance to biotic stresses, which have not been widely utilized or incorporated into modern varieties (Ram et al., 2007). The landraces grown in rice blast “hot- spots” of the Indian sub-continent has remained largely unexplored. Molecular markers linked to major *R*-genes represent an important tool for marker assisted selection (MAS) (Costanzo and Jia, 2010). Variations in terms of Single Nucleotide Polymorphisms (SNPs) and insertion-deletions (InDels) covering the entire genic segment can be compared among genotypes to identify functional markers to aid the selection process. Markers associated with two cloned blast *R* genes (*Pi-b* and *Pi-ta*) as well as a PCR-based SNP markers for *Piz* locus and *Pik* locus (Hayashi et al., 2004; Jia et al., 2009; Zhai et al., 2011) are to mention a few. Conventional breeding with MAS would therefore, benefit from the development of new *R* gene specific markers, which would allow pyramiding of multiple genes in adapted germplasm toward realizing broader spectrum disease resistance. Molecular population genetic analysis of local landraces and cultivated varieties might provide insight on the selection forces maintaining resistance and preventing evolution of new specificities in natural pathogen populations. Therefore, this study was conducted with objectives, (i) analysis of variants of *Pi54* alleles from the cultivated varieties and Indian landraces of rice collected from different eco-geographical regions (ii) structural analysis of *Pi54* alleles to understand molecular evolution at the loci, and (iii) development of allele specific functional markers for use in marker assisted selection.

## Materials and methods

### Plant material and fungal culture

A set of 92 rice lines (landraces and cultivated varieties) were selected from different geographic locations of India for prospecting of *Pi54* alleles. The diagnostic isolate of *M. oryzae* (Mo-nwi-37-1) was used for the phenotypic evaluation of all the rice lines (Rai et al., 2011; Rathour et al., unpublished data).

### Preparation of fungal culture

Fungal culture of Mo-nwi-37-1 was maintained on Oat Meal Agar (HiMedia, India) medium in pre-sterilized pertiplates (90 mm diameter). For sporulation, the culture was multiplied in Mathur's medium (Dextrose 8 g/L, Magnesium sulfate 2.5 g/L, Potassium phosphate 2.75 g/L, Neo-Peptone 2.5 g/L, Yeast Extract 2.0 g/L, and agar 16 g/L). The culture plates were maintained at 22°C for 12–16 days under constant illumination with white fluorescent light (55 μF/Em/s). For the preparation of fungal spores, 5 ml of 0.2% gelatine solution was added to individual plate on agar surface and gently rubbed with scrapper to separate conidia from the conidiophores. The spore concentration was brought to approximately 10^5^ spores/ml. The seedlings were sprayed with spore suspension of about 1 ml per plant at 2–3 leaf stage.

### Inoculation of rice lines with diagnostic *M. oryzae* isolate

Rice lines were grown in plastic pots (12 inch dia.) containing sterilized potting mixture in the rice blast testing facility, NRCPB, IARI, New Delhi. Rice lines, Tetep, and Taipei 309 were used as positive and negative controls, respectively. Physical parameters were set for 16 h/8 h light-dark photoperiod. The day and night temperatures were maintained at 25°C and 21°C, respectively, with relative humidity (RH) of more than 90%. All the seedlings assessed in the experiment were sprayed simultaneously with *M. oryzae* spore suspension of 10^5^ spores/ml. Disease reaction was recorded after 7 days of inoculation using 0–5 disease assessment scale (Bonmann et al., 1986). Where, 0 = No evidence of infection; 1 = Brown specks smaller than 0.5 mm in diameter, no sporulation; 2 = Brown specks about 0.5–1.0 mm in diameter, no sporulation; 3 = Roundish to elliptical lesions, 1–3 mm in diameter, gray center surrounded by brown margins, lesions capable of sporulation; 4 = Typical spindle shaped blast lesions capable of sporulation, 3 mm or longer; 5 = lesions as in 4 but about half of 1–2 leaf blades killed by coalescence of lesions. Reaction types 0, 1, 2, and 3 were considered resistant, while 4 and 5 considered as susceptible.

### PCR amplification and sequencing

Genomic DNA was extracted from fresh leaves of selected rice lines using the modified Cetyltrimethyl Ammonium Bromide (CTAB) method of DNA isolation (Murray and Thompson, 1980). For PCR amplification, nucleotide sequence of the blast resistance gene *Pi54* (Loc_Os11g42010) was retrieved from NCBI database (www.ncbi.nlm.nih.gov/). Overlapping oligos Pi54_F1 (CAATATAGCTGGGAATTTCAGAGG) and Pi54_R1 (AGATAATGTGTTTGTCTGGCTGTC); Pi54_F2 (CATGAACAGAGCACTGATGACATA) and Pi54_R2 (GGATAACAAGCACTGAGCCATATC); Pi54_F3 (CCGTTCTGACCATAGAAATTATCG) and Pi54_R3 (GTGCAATTACATAAGCTAGACCTTG) were designed using Primer 3 software (Rozen and Skaletsky, 2000) to amplify 1.5 kb region using primer walking technique. PCR was performed with genomic DNA isolated from rice landraces and cultivated varieties using Pfu polymerase (FINNZYMES OY, Keilaranta, Espoo, Finland) with the following thermal cycling conditions: initial DNA denaturation at 95°C for 2 min followed by 30 cycles of 95°C for 30 s, 58°C (Pi54_F1 and Pi54_R1; Pi54_F2 and Pi54_R2) or 60°C (Pi54_F3 and Pi54_R3) for 30 s, 72°C for 1 min, final elongation at 72°C for 10 min and hold at 4°C. The PCR derived amplification products were used as template for determining DNA sequences using Sanger‘s dideoxy method of sequencing.

### Sequencing of PCR amplicon

The purified PCR amplicon was sequenced according to manufacturer's instruction directly by using modified Sanger's dideoxy terminator cycle sequencing chemistry on an automated capillary-based DNA sequencer (ABI 3730xl DNA Analyzer) in both forward and reverse direction twice using amplified product specific primers. The PCR products were run in a cycle sequencing reaction with thermal cycling conditions as 30 cycles of denaturation (95°C for 20 s), annealing (60°C for 20 s), and extension (60°C for 4 min) followed by hold at 4°C. The purified sequencing products were resolved on a capillary-based automated DNA sequencer (ABI 3730xl DNA Analyzer). Full length sequence reads were obtained by assembly of multiple reads of each fragment using Phred/Phrap and Consed software (Ewing and Green, 1998). Each fragment was sequenced at least four times and high quality (Phred 20) consensus sequence was used for data analysis.

### Analysis of sequenced *Pi54* alleles

The sequenced data were aligned using ClustalW 2.0 (Larkin et al., 2007) at their default alignment parameters and manually corrected by MEGA 4.0. Gene coding regions were predicted with FGENESH (Solovyev et al., 2006) using the original *Pi54* (Tetep) sequence as a reference. The LRR domain was identified as described earlier (Sharma et al., 2005a). Motif was identified using motif scan software (http://hits.isb-sib.ch/cgi-bin/PFSCAN) and SMART tool (http://smart.embl-heidelberg.de/). Phylogenetic analysis was performed with MEGA 4.0 using the Neighbor-Joining method (Saitou and Nei, 1987). The bootstrap consensus tree inferred from 1000 replicates was used to represent the evolutionary history of the taxa analyzed (Felsenstein, 1985). Branches corresponding to partitions reproduced in less than 50% bootstrap replicates were collapsed. The tree was drawn to scale, with branch lengths in the same units as those of the evolutionary distances used to infer the phylogenetic tree. The evolutionary distances were computed using the Maximum Composite Likelihood method (Tamura et al., 2004) and were depicted in the units of the number of base substitutions per site. All positions containing gaps and missing data were eliminated from the dataset (Complete deletion option). Analysis of overall transition: transversion ratio, variable, and parsimony informative positions were calculated using MEGA 4.0 software (Tamura et al., 2007).

The DnaSP 5.10 software was used for the analysis of nucleotide polymorphism (Rozas et al., 2003). The aligned DNA sequences were imported into the DnaSP software to calculate S (number of polymorphic or segregating sites), π (nucleotide diversity), θ (Theta from S, Theta-W), and D (Tajima's D), and to draw the sliding window of nucleotide diversity (π). Haplotype networks were constructed for each potential SNP's sites by statistical parsimony with the software TCS 1.21 (Templeton et al., 1992; Clement et al., 2000). The networks were assembled based on an absolute distance matrix between haplotypes, i.e., the number of mutations separating each haplotypes, with a parsimony probability of 95%. Haplotype diversity (Hd) was analyzed between disease resistance and susceptible phenotypes of *Oryza* species. The DnaSP 5.10 program (Rozas et al., 2003) was used for the analysis of haplotype diversity Hd (Nei, 1987).

### Development of functional markers

The PCR-based co-dominant and dominant STS (sequence tagged site) markers targeting consensus InDels of 144 and 163 bp, respectively were designed. In case of dominant marker, the forward primer (DPi54_163F) was designed flanking to the insertion (163 bp) and reverse primer (DPi54_163R) was designed from the sequence of insertion. The forward (CPi54_144F) and reverse (CPi54_144R) primers in case of co-dominant markers were designed from the flanking regions of 144 bp insertion. Primer pairs [CPi54_144F (AAGTACTTCATGATCTATTCTACTGG) and CPi54_144R (CCGTTCTGACCATAGAAATTATCG)]; DPi54_163F (ACCATGACTAGCTATGAAAAATCT) and DPi54_163R (AGAATAGATCATGAAGTACTTGAAAC)] were designed by using Primer 3 software (Rozen and Skaletsky, 2000). PCR amplification was carried out on programmable Thermal Cycler (BioRad, Washington DC, USA) using the following temperature profile: initial DNA denaturation, 95°C for 2 min; followed by 35 cycles of denaturation, 94°C for 20 s; annealing, 55°C (DPi54_163F and DPi54_163R) or 58°C (CPi54_144F and CPi54_144R) for 30 s; extension, 72°C for 1 min; and final extension at 72°C for 10 min and then hold at 4°C using Taq DNA polymerase (Vivantis, USA). The PCR amplified product was resolved in 2% agarose gel using 1.0X TAE buffer.

## Results

### Phenotypic evaluation of rice landraces

The rice lines (92) used in present study were grown in contained condition and the 15 days-old-seedlings were challenged with representative *M. oryzae* isolate, Mo-nwi-37-1. After a week of inoculation, all the rice lines were grouped into resistant and susceptible categories based on their reaction to *M. oryzae*. Out of 92 rice lines, 72 were found resistant and the rest 20 susceptible, based on disease assessment scale (Bonmann et al., 1986) (Table S1). These lines were used for the allele mining studies of *Pi54* gene.

### Nucleotide polymorphism

**Figure 1:**
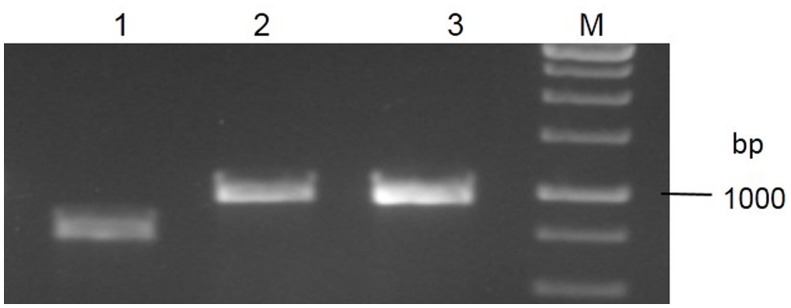
**PCR amplification of different fragments of ***Pi54*** allele of rice landrace, Tiyun**. bp- Base pairs, M- Molecular weight marker and lanes 1–3, PCR fragments obtained from overlapping fragments of *Pi-54* allele.

To determine the nucleotide diversity at the *Pi54* locus in rice lines, 1.5 kb long fragments were amplified from 92 rice lines and sequenced using 3 different overlapping primer combinations (Figure 1). All the fragments were sequenced and high quality (>Phred 20) assembled sequence of each allele has been deposited in the EMBL database (Table S1). For sequence analysis, the *Pi54* alleles were grouped into three different categories: (i) phenotypes: resistant and susceptible (ii) landraces and cultivated varieties; (iii) *indica* and *japonica* types (Table 1). Nucleotide variations were high in the genic region of *Pi54* allele. A total of 197 SNPs, large insertions of 38, 49, 144, and 163 bps and single base pair deletions were identified in the *Pi54* alleles.

**Table 1:** **Different groups and rice accessions used in mining for blast resistance gene ***Pi54*****.

**Group**	**Species**	**No. of accessions**
Cultivar	*O. sativa indica*	48
	*O. sativa japonica*	3
Landrace	*O. sativa indica*	32
	*O. sativa japonica*	3
	*O. sativa aus*	2
Breeding line	*O. sativa indica*	2
Hybrid	*O. sativa indica*	1
Unknown	*O. sativa indica*	1

**Figure 2:**
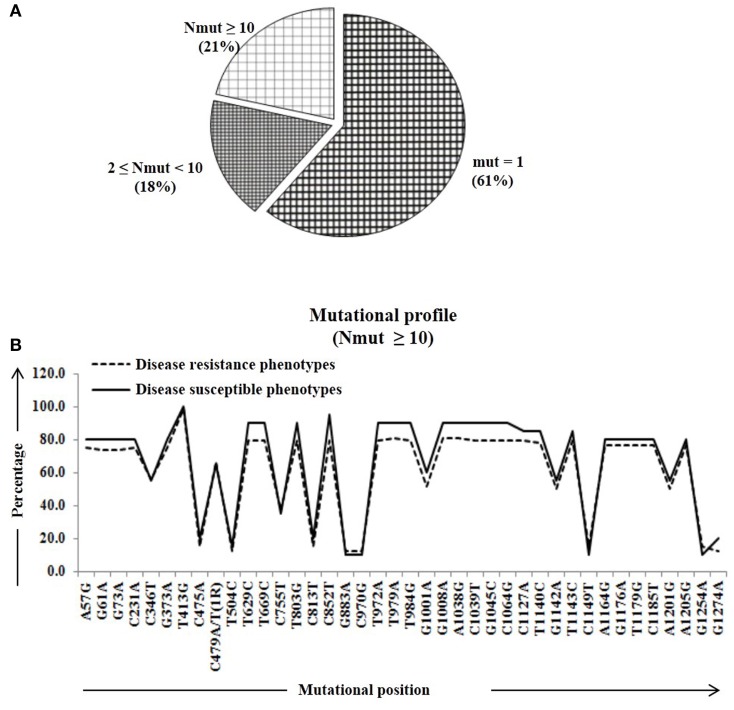
**The number of amino acid substitutions in the predicted 64 Pi54 protein**. **(A)** Distribution of amino acid substitutions across 64 predicted proteins. **(B)** Substitutional profile of amino acids of disease resistant and susceptible phenotype.

We calculated percentage of mutational change with respect to the reference *Pi54* gene and compared within and between disease resistance and susceptible alleles of *Oryza* species. All mutational changes were scored for the specific positions only. The number of mutations per site was found to be equal to and greater than 10 (i.e., N_mut_ ≥ 10). Overall, 40 mutational sites were identified across the alignment, out of which 27 are transitions (ti) and 12 transversion (tv) (Table S2). Further, mutational profiling of disease resistance and susceptible phenotypes of *Oryza* species was constructed in all alleles mined from 92 rice landraces. It was found that 61% of mutational sites have mutations at one site, 18% of the sites have mutations at two-nine sites and rest of the 21% sites have mutation at 10 or more than 10 sites (Figure 2A). Most of the mutational sites exhibited a constant frequency distribution between the resistance and susceptible groups (Figure 2B).

**Figure 3:**
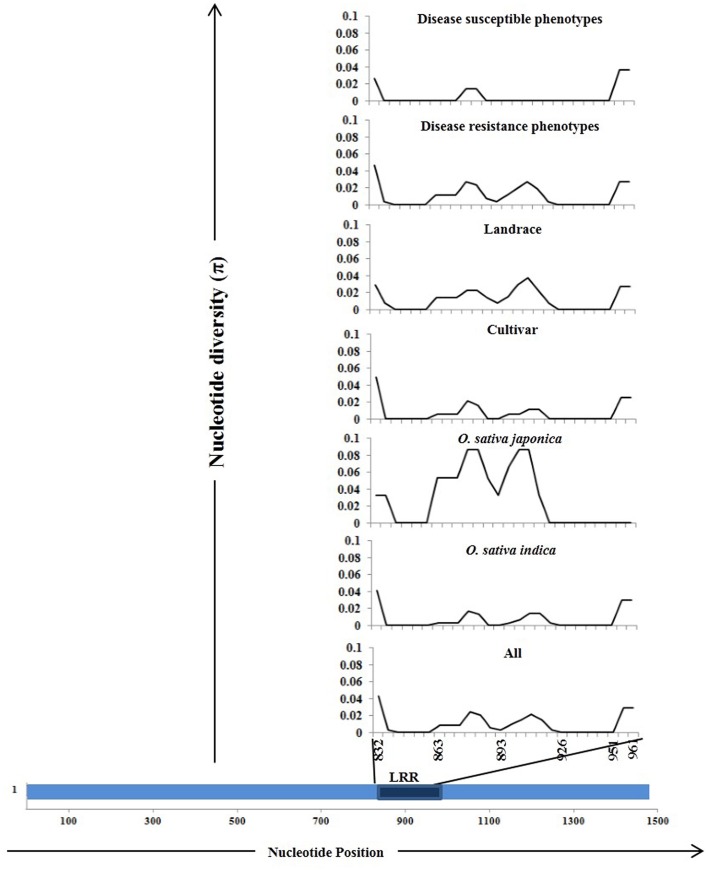
**Sliding window analysis of nucleotide diversity (π) in the LRR domains of 92 ***Pi54*** alleles**. The different groups from where the alleles were mined are given above in the Figure. The nucleotide diversity (π) was plotted on Y-axis and the X-axis represents the positions of nucleotides.

The *Pi54* alleles of landraces harbor substantially higher polymorphism as compared to the *Pi54* alleles of cultivated species, because of heterogeneous nature of rice landraces used in present study, which might have accumulated more mutations during the course of evolution. The nucleotide diversity was high in the *Pi54* alleles of resistant lines compared to the *Pi54* alleles of susceptible lines (Table 2). The *Pi54* alleles of *indica* and *japonica* species were almost equally diverse at nucleotide level. Higher nucleotide variation at the resistant loci is possibly due to interaction with highly avirulent and frequently mutating avirulent strains of *M. oryzae*. Overall, a total of 198 polymorphic sites excluding InDels, were identified in the 1.5 Kb region of *Pi54* alleles. Maximum (225) polymorphic sites were identified in the *Pi54* alleles isolated from Indian landraces; however, very less diversity was obtained in the alleles cloned from *japonica* species (Table 2). Average pair wise nucleotide diversity (π) and Watterson's nucleotide diversity estimator (θ_w_) over the *Pi54* alleles in susceptible rice lines (π = 0.0208 and θ_w_ = 0.01916) was lowest among all other *Pi54* alleles included in the present study. Within the groups, the nucleotide diversity was lowest in *Pi54* alleles of cultivated varieties (π = 0.02254 and θ_w_ = 0.02102) compared to landraces (π = 0.03417 and θ_w_ = 0.03877). Among the *Pi54* alleles of *indica* and *japonica* species, diversity was low in the alleles of *japonica* species (Table 2). The LRR domain harbors substantial diversity, explaining the selection pressure it underwent. Higher diversity was observed in LRR domain of *japonica* species as compared to others (Figure 3).

**Table 2:** **Nucleotide polymorphism of the ***Pi54*** alleles mined from different accessions of rice**.

**Group**	**Elements**	**Position (nt)**	**S**	**π**	**P(π)**	**Θ**	**P(Θ)**	**Tajima's D**	**Ka/Ks**
All	Whole sequence	[1–4194]	198	0.0226	0.00146	0.02972	0.00801	−1.07559	<1
	CDS	[1338–3585]	163	0.02541	0.0018	0.03289	0.00893	−1.03898	<1
	LRR	[3324–3462]	15	0.01073	0.00218	0.0235	0.00847	**−1.70363**	>1
*O. sativa indica*	Whole sequence	[1–4194]	179	0.02245	0.00131	0.02717	0.00753	−0.87382	<1
	CDS	[1338–3585]	140	0.02431	0.00149	0.029	0.00811	−0.86041	<1
	LRR	[3324–3462]	9	0.00839	0.00123	0.01451	0.00607	−1.34456	>1
*O. sativa japonica*	Whole sequence	[1–4194]	77	0.0238	0.00487	0.02285	0.01094	−0.06202	<1
	CDS	[1338–3585]	68	0.02899	0.00632	0.02815	0.01352	−0.08869	<1
	LRR	[3324–3462]	9	0.02815	0.01044	0.0292	0.01615	−0.21398	>1
Cultivar	Whole sequence	[1–4194]	123	0.02254	0.00144	0.02102	0.00646	−0.08589	<1
	CDS	[1338–3585]	106	0.02576	0.00176	0.02431	0.00752	−0.14919	<1
	LRR	[3324–3462]	7	0.00883	0.00153	0.01259	0.00587	−1.13434	>1
Landrace	Whole sequence	[1–4194]	225	0.03417	0.00467	0.03877	0.01246	−0.94232	<1
	CDS	[1338–3585]	141	0.02451	0.00371	0.03502	0.01139	−1.36704	<1
	LRR	[3324–3462]	13	0.01247	0.00461	0.02524	0.01036	**−1.72346**	>1
Disease resistance phenotypes	Whole sequence	[1–4194]	192	0.0237	0.00171	0.03004	0.00848	−0.9818	<1
	CDS	[1338–3585]	158	0.0267	0.00215	0.03366	0.00957	−0.96141	<1
	LRR	[3324–3462]	15	0.01213	0.00267	0.02481	0.00917	**−1.67675**	>1
Disease susceptible phenotypes	Whole sequence	[1–4194]	87	0.0208	0.00286	0.01916	0.00728	0.27313	<1
	CDS	[1338–3585]	71	0.02208	0.00327	0.02104	0.00806	0.15424	<1
	LRR	[3324–3462]	3	0.0057	0.00174	0.00699	0.00454	−0.56505	>1

*S, Number of Polymorphic or Segregating sites; π, Nucleotide diversity; Θ, Watterson's nucleotide diversity estimator based on silent sites. Tajima's D calculation based on the difference between the number of segregating sites and the average number of nucleotide differences. Jukes–Cantor (JC) corrected synonymous differences per synonymous site (Ks) and non-synonymous differences per non-synonymous site (Ka). For Tajima's D, no value is significant at 0.10 level (P > 0.10) and 0.10 > P > 0.05 (for bold)*.

### Phylogenetic relationship between the alleles

**Figure 4:**
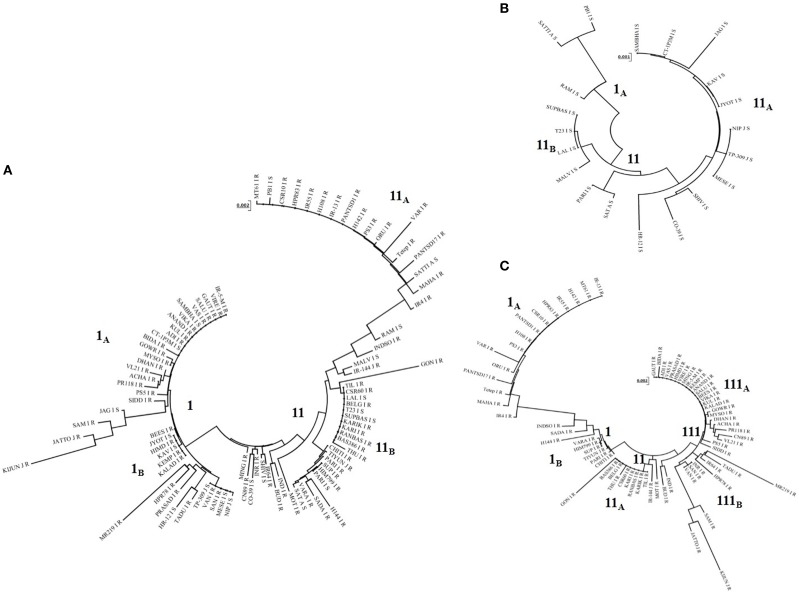
**Phylogenetic analysis of ***Pi54*** alleles mined from different rice accessions**. **(A)** Phylogenetic tree of *Pi54* alleles based on the nucleotide sequences of 92 rice accessions. **(B)** Phylogenetic tree of *Pi54* alleles based on the nucleotide sequences of 20 blast susceptible rice accession. **(C)** Phylogenetic tree of *Pi54* alleles based on the nucleotide sequences of 72 blast resistant accessions. N-J method was used for the construction of tree. No definite clustering was obtained in all the trees.

Phylogenetic tree constructed based on *Pi54* allelic sequences obtained from 92 accessions resulted in two major clusters (Figure 4). Both the clusters were further divided into separate sub-clusters but species specific clustering was not obtained. Similarly, separate phylogenetic tree was also constructed for the *Pi54* alleles derived from resistant and susceptible lines. All the resistant *Pi45* orthologs were grouped into three clusters (Figure 4A). Cluster I and II were further divided into two sub-clusters, i.e., I_A_, I_B_, and II_A_, II_B_. The sub-cluster I_A_ and cluster III consisted of all the resistance alleles belonging to *indica* group whereas all other sub-clusters included alleles of *indica* as well as *japonica* groups. The *Pi54* alleles from susceptible lines were grouped into two major distinct clusters (Figure 4B).

### Pattern of molecular evolution

**Figure 5:**
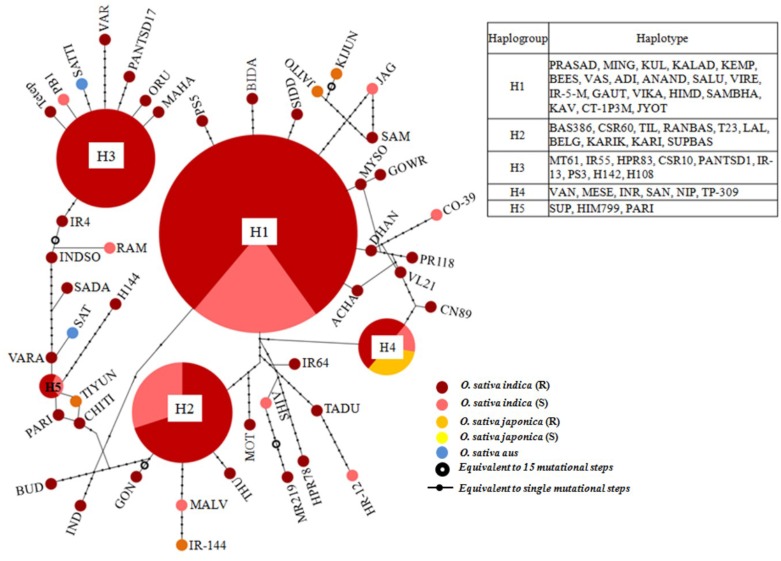
**A haplotype network based on potential SNPs of the 92 ***Pi54*** alleles**. Each group of haplotypes is shown as a solid circle, and five major haplotypes are marked in larger circles. Each branch represents a single mutational step. Branches with small solid circles indicate that there is more than a single mutational step between haplotypes. Different sizes of circles represent the different numbers of each haplotype.

A haplotype network was constructed to identify mutational changes, based on potential SNPs of all *Pi54* alleles isolated from 92 rice lines. We identified fifty new haplotypes from the nucleotide polymorphism of the *Pi54* alleles. To determine the linkage among these haplotypes, a haplotype network was constructed (Figure 5). In this network, 50 haplotypes were clustered in five major haplogroup (major haplogroup contain three or more *Pi54* alleles) and the rest as minor haplogroups. We identified resistant phenotype specific haplotype (H_3) and rest of the major haplotypes contained alleles from resistant and susceptible lines as well. Furthermore, haplotype, H_4 was the only mixed haplotype consisted of the *Pi54* alleles of *indica, japonica* and *aus* rice accessions. Similar haplotype network has been constructed for resistant as well susceptible *Pi54* alleles. In case of resistance alleles, total number of identified haplotypes was 41 which clustered into five major haplogroups (Figure S1). All the major haplogroups consisted of *Pi54* alleles isolated from *indica* rice lines. Total number of 14 haplotypes identified in *Pi54* alleles was from susceptible rice lines and were, clustered into four major haplogroups (Figure S2). The major haplogroup of these rice lines consisted of *Pi54* alleles of *Indica* rice lines except for H1 which is a mixed cluster of alleles from both *indica* and *japonica* lines. Statistically high haplotypes diversity (0.935/0.019) was observed within the studied data set of 92 *Pi54* alleles of *Oryza* species. In case of susceptible and resistant alleles, high haplotypes diversity (0.958/0.028) was observed in disease susceptible *Pi54* alleles of *Oryza* species than resistance (0.935/0.019) alleles (Table 3).

**Table 3:** **Summary statistics of haplotype diversity in ***Pi54*** alleles**.

**Group**	**Hn**	**Hd**	**V(Hd)/SD**
All	50	0.935	0.00027/0.016
Disease resistance phenotypes	41	0.935	0.00036/0.019
Disease susceptible phenotypes	14	0.958	0.00079/0.028

*Hn, Number of Haplotypes; Hd, Haplotype diversity; V(Hd), Variance of Haplotype diversity; SD, Standard deviation of Haplotype diversity*.

In present study, sequence analysis indicated variable number (0–3) of Open Reading Frames (ORFs) in the *Pi54* locus, with exception of 28 rice lines where no ORF was detected. Absence of ORF in these sequences might be due to reshuffling or recombination events of the locus resulting in absence of start codon or pseodogenized allele, which has lost its function in due course of time. In 40 rice lines, single exon was predicted. However, two and three exons were also predicted in the allelic sequences of 21 and 10 rice lines, respectively (Table S3). This may be due to the creation of new splice sites during the course of evolution. Variation in the number of ORF might have generated based on the selection pressure, it underwent during the evolutionary process. Various insertions have also been identified in the ORFs. The presence of insertion implicates its differential role in regulating disease resistance.

**Figure 6:**
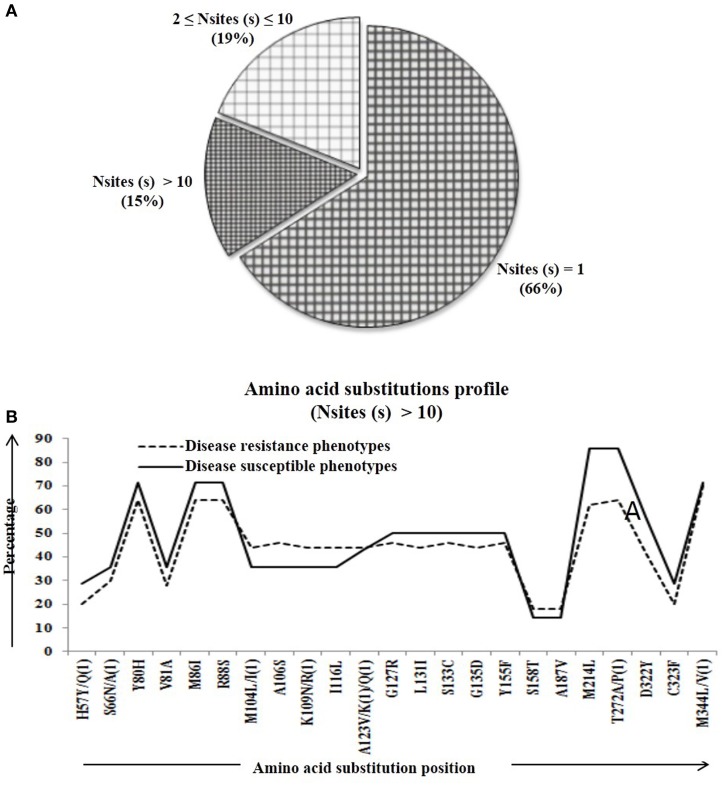
**The number of amino acid substitutions in the predicted 64 Pi54 protein**. **(A)** Distribution of amino acid substitutions across 64 predicted proteins. **(B)** Substitutional profile of amino acids of disease resistant and susceptible phenotype.

Percentage of substitutional change was calculated at protein level in the *Pi54* gene and compared within and between disease resistance and susceptible phenotypes of *Oryza* species. The amino acid substitutions were scored for the specific positions. The number of substitution per site was found to be equal to and greater than 10 (i.e., N_mut_ ≥ 10). Overall, 23 substitutional sites were identified across the alignment of 64 Pi54 proteins (Table S4). The number of mutations per mutational site was calculated in all the aligned 64 predicted sequences implicating 66% of mutational sites having mutation in one site, 19% of the sites having mutations in two to nine sites and rest of the 15% sites having mutation in 10 or more than 10 sites (Figure 6A). The mutational profiling of disease resistance and susceptible phenotypes of *Oryza* species indicated that most of the mutational sites were showing a constant frequency distribution between the resistance and susceptible groups (Figure 6B).

The nucleotide sequences of all the *Pi54* alleles were translated and the predicted proteins ranged between 73 and 486 amino acid residues having many predicted functional domains. The Zinc-finger domain (ZnF) was predicted in all the Pi54 proteins except for a few (Figure S3). The sequence of predicted ZnF domain was highly conserved (100% similarity) in all the Pi54 proteins (Figure S3). Important motifs identified in the translated sequences of the *Pi54* alleles are N-glycosylation sites, phosphorylation (kinase C phosphorylation site, casein kinase II phosphorylation site, tyrosine kinase phosphorylation site), tyrosine sulfation site, N-myristoylation site, and leucine zipper (Table S5). These sites were present in variable numbers in all the *Pi54* alleles except for tyrosine kinase phosphorylation site and leucine zipper. The presence of phosphorylation sites in *Pi54* alleles, indicate their involvement in signal transduction by activating further downstream genes. The presence of N-myristoylation sites in the predicted proteins play important role in membrane anchoring whereas N-glycosylation sites has significant role in protein targeting. Unique leucine zipper was identified in 54 and 73% of the resistant and susceptible Pi54 proteins, respectively. They are usually found as a part of DNA-binding domain in many transcription factors, and are therefore involved in regulating gene expression. Similarly, tyrosine sulfation sites were also present in 18 and 21% of the resistant and susceptible Pi54 protein, respectively, which plays important role in strengthening the protein-protein interaction. From above results, it can be concluded that unique Leucine zipper and single tyrosine sulfation sites identified in some of the Pi54 predicted protein sequences which was absent in the reference Pi54 protein.

To evaluate the phylogenetic relationship amongst the predicted Pi54 proteins, neighbor-joining trees were constructed using the LRR regions (Figure S4). All the predicted Pi54 proteins were grouped into three separate clusters of mixed type. This is in contrast to species-specific groups obtained from NBS and LRR domains of *Pi9* alleles (Liu et al., 2011).

### Analysis of evolutionary dynamics

To test the evolutionary selection dynamics of the *Pi54* alleles in 92 *Oryza* accessions, we evaluated the extent of neutral selection with D statistics (Tajima's D test) (Tajima, 1989). In the present study, *Pi54* alleles have been subjected to positive selection [Tajima's D = −1.07559] for all the alleles and deviating from the model of neutrality (Table 2). It is noteworthy that *Pi54* alleles of different groups, such as landraces and cultivated varieties, *indica* and *japonica* species, and blast resistant lines have been subjected to positive selection whereas the balancing selection operates in *Pi54* alleles of susceptible rice lines [Tajima's D = 0.27313]. This might be due to the variable selection pressure acting on the locus, or diverse sample size used in present analysis. Further, coding region of 92 *Pi54* alleles and *Pi54* alleles of different groups have been analyzed. The value of Tajima's D was negative in the entire coding region (−1.03898) and LRR domain (−1.70363) indicating purifying selection in the CDS and LRR regions (Table 2). The ratio of synonymous (k_s_) and non-synonymous (k_a_) divergence in whole sequence as well as coding region and parts of the coding region (LRR domains) was calculated in all the 92 *Pi54* alleles and separately for the *Pi54* alleles from different groups (Table 2). The value of k_a_/k_s_ ratio was used as a criterion for the presence or absence of positive selection for amino acid substitutions. The value of k_a_/k_s_ in the whole sequence, coding region of the *Pi54* alleles was less than one, indicating low level of polymorphism in these regions, in contrast to high level of polymorphism in LRR domain of all *Pi54* alleles as the ratio of k_a_/k_s_ was greater than one (Table 2). In the LRR region of the *Pi54* alleles of different groups, the value of k_a_/k_s_ is greater than one, which indicates that positive directional selection might have favored amino acid substitution in this region. In the present study, LRR region of *Pi54* alleles was quite variable and might have role in different recognition specificities, possibly making it more durable.

### Development of allele specific markers

**Figure 7:**
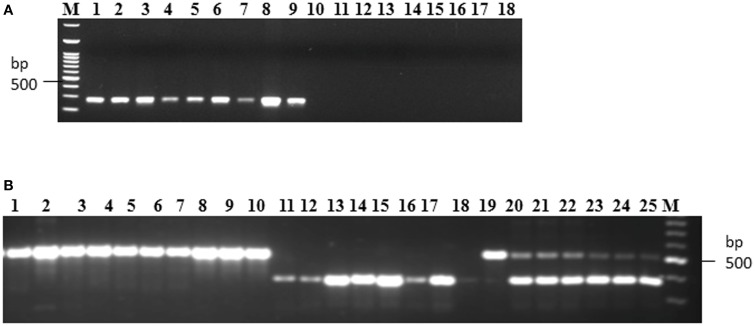
**PCR amplification of rice lines with the dominant and co-dominant PCR marker**. **(A)** PCR amplification with the dominant marker (D163_F and D163_R) in 18 rice lines. Lanes 1–9: presence of band in rice lines (Acharmati, Aditya, Ananda, Beesginsali, Bidarlocal-2, Dhanaprasad, Gautam, Himdhan, and Pusa sugandh 5); lanes 10–18: absence of band in rice lines.(HR-12, TP-309, Co-39, Nipponbare, Shiva, Satti, Pusa basmati-1, Ram Jawain-100, and Sathia-2) **(B)** PCR amplification with the co-dominant marker (C144_F and C144_R) in 26 rice lines. bp- base pair; M- 100 bp molecular marker. Lanes 1–10: presence of susceptible band (CO-39, HR-12, CT-10006-7-2M-5-1P3M, Jyoti, Kavali kannu, Mesebatta, Nipponbare, Samba mahsuri, Shiva, Taipei-309); lanes 11–18: presence of resistant band (Tetep, Budda, Chiti zhini, CSR 10, Gonrra bhog, Himalya 799, Pusa Sugandh 3, Ranbir basmati, Sadabahar); lanes 19–25: presence of both the bands (Belgaum basmati, CSR-60, HLR-142, Indrayani, IRAT-144, Kariya).

Allele specific DNA markers are important for the introgression of resistant alleles in cultivated rice varieties using marker assisted selection (MAS) strategy. In this study, we developed dominant and co-dominant STS markers based on the large DNA insertions in the allelic sequences of *Pi54* alleles. The dominant markers (DPi54_163F and DPi54_163R) were specifically designed to amplify a fragment of 278 bp in resistant *Pi54* alleles and absence of band in the susceptible alleles (Figure 7A). By using this marker, we were able to distinguish 22 rice lines having resistant *Pi54* allele (presence of 278 bp amplification product) and 15 rice lines having susceptible *Pi54* alleles (Table 4). Similarly, co-dominant marker (CPi54_144F and CPi54_144R) was also tested in a set of rice lines used in present study. PCR amplification with co-dominant markers amplifies fragments of 557 and 313 bp (Figure 7B). The 557 bp fragments were amplified in 10 susceptible rice lines, and a 313 bp band was present in 28 resistant rice lines. Eight rice lines showed presence of both the alleles (Table 5).

**Table 4:** **List of rice accessions distinguishable based on dominant marker**.

**Homozygous RR**		**Homozygous rr**
Acharmati	Pusa sugandh 5	Mesebatta
Aditya	Salumpikit	HR-12
Ananda	Samleshwari	TP-309
Beesginsali	Siddasala	Co-39
Bidarlocal-2	V L Dhan 21	Nipponbare
Dhanaprasad	Vasanesanna batta	Shiva
Gautam	Vikash	Satti
Himdhan	Virendra	Pusa basmati-1
IRBB-4		Ram Jawain-100
IRBLB-BIRBLB-5-M		Sathia-2
Kala Dhan		Lalan kanda
Kulanji pille		Superbasmati
Mingola		Parijat
Mysore mallige		Malviya dhan
		T23

**Table 5:** **List of rice accessions distinguishable based on co-dominant marker**.

**Homozygous (RR)**		**Homozygous (rr)**	**Heterozygous (Rr)**
Budda	Mahamaya	CO-39	Belgaum basmati
Chiti zhini	MTU-1061	CT-10006-7-2M-5-1P3M	CSR-60
CSR 10	Pant sankar dhan 1	HR-12	HLR-142
Gonrra bhog	Pant sugandh dhan 17	Jyoti	Indrayani
Himalya 799	Tetep	Kavali kannu	IRAT-144
HLR-108	Pusa Sugandh 3	Mesebatta	Kariya
HLR-144	Ranbir basmati	Nipponbare	Orugallu
HPR 2083	Sadabahar	Samba mahsuri	Parimala kalvi
HPR-2178	Suphala	Shiva	
Indira sona	Thule atte	Taipei-309	
IRBB 55	Tilak chandan		
IRBB-13	Tiyun		
IRBB-4	Varalu		
Kari kantiga	Varun dhan		

## Discussion

Breeding efforts have capitalized only a fraction of the genetic diversity available to us. Food availability needs to be increased in face of intensifying demand, climate change, soil degradation, land, and water shortages. Farmers are saviors of seeds of crop species, primitive varieties (local domesticates called *landraces*), wild relatives of crop species (McCouch, 2013). The biodiversity present within the farmer adapted land races must be mined to discover novel sources of resistance to pests and diseases. Chromosome 11 of rice as reported, has the most associated disease resistance loci and the highest frequency of copy number variations (CNVs). Genes in most of CNVs were reported to be associated with resistance phenotype (Yu et al., 2011; Wang et al., 2014). The allelic variants of the dominant blast resistance gene, *Pi54* located on chromosome 11 were prospected and variations in terms of SNPs and InDels were documented. These variations possibly might play an important role in the durability of *Pi54* gene against *M. oryzae* population. In earlier studies, InDels and SNPs have shown to play a pivotal role in *R*-gene evolution through selection (Shen et al., 2006). The presence of 5 Mb region (super locus) physically linked to *Pi-ta* gene impart resistance phenotype (Jia and Martin, 2008; Lee et al., 2009). The higher frequency of SNP observed in present study might be due to the combined analysis of both landraces and cultivated species. Similarly, higher variation was also observed between *O. sativa* and *O. rufipogon* in 26 kb region of DNA sequence spanning 22 loci (Rakshit et al., 2007). Further in all the *Pi54* alleles, transitions were more frequent than transversions. This complies with the common composition of any type of DNA, where transitions have been reported to occur at higher frequencies than transversions (Brown et al., 1982; Gojobori et al., 1982; Curtis and Clegg, 1984; Wakeley, 1996). This is in consistent with the earlier genome wide SNP discovery studies in multiple rice genotypes (Huang et al., 2009; McNally et al., 2009; Yamamoto et al., 2010; Thakur et al., 2014). Relatively higher (70%) frequency of transition substitutions between *indica* and *japonica* was observed in earlier studies (Feltus et al., 2004; Shen et al., 2004; International Rice Genome Sequencing Project, 2005). The present study indicates that *Pi54* alleles belong to type II category (intermediate diversified), similar to other blast resistance gene *Pi 9* (Yang et al., 2008; Liu et al., 2011). It is increasingly believed that percent polymorphism is directly correlated to evolutionary change (Shen et al., 2006; Yang et al., 2008). Our results suggest that intermediate level of polymorphism in the *Pi54* alleles may be due to the mixed evolutionary pressure experienced by the loci during co-evolution of rice blast pathogen. Since this gene has not been transferred to cultivated varieties and might have less pressure from pathogen side.

In present study, 198 polymorphic sites were identified in the 1.5 Kb region of *Pi54* alleles. The nucleotide diversity was high in the *Pi54* alleles of resistant lines compared to the susceptible alleles. Previously, it was reported that *R*-genes experience both high and low levels of sequence diversity depending upon the locus (Yang et al., 2008). Nucleotide diversity (0.024) in *A. thaliana* was higher than the average π (0.008) in 334 randomly distributed genomic regions due to nucleotide difference between resistant and susceptible alleles indicating that these alleles have been maintained for long period of time under natural conditions (Schmid et al., 2005). In barley, the frequency and distribution of the nucleotide diversity ranged from 0.0021 to 0.0189 for the genes associated with grain germination (Russell et al., 2004). In another study, the pattern of diversity observed was lowest in cultivated species as compared to other *Oryza* species (Lee et al., 2009). However, the variation at the flanking regions of the *Pi-ta* gene was highest in *O. rufipogon* (0.00355) followed by cultivated species of *Oryza* (Lee et al., 2011). Studies of many *R* gene loci, such as *Rpp5* in *Arabidopsis thaliana* and *Rp1* in *Zea mays* (Noel et al., 1999; Sun et al., 2001) have revealed a high level of polymorphism between the alleles. In resistance landraces, the expression of Os11g0225100 locus was higher compared to susceptible. Even after inoculation, the resistance level in the resistant landrace increased, while it has no change in the susceptible landrace. This high diversity is interpreted as evidence for the fast evolution of these *R* gene loci.

Phylogenetic analyses of the *Pi54* alleles from susceptible lines were grouped into two major clusters, while all the resistant *Pi45* orthologs were grouped into three clusters. In all the clusters and sub-clusters mixed type of grouping was obtained. However, landraces are found in all the sub-clusters corroborating the claim of higher variability and heterogeneity. This is in contrast to *Pi9* alleles wherein cultivated rice along with its ancestors clustered into one group and African cultivated rice along with its ancestors grouped into separate cluster suggesting that different selection pressure has occurred in two groups during domestication and/or natural selection (Liu et al., 2011).

Important motifs identified in the translated sequences of the *Pi54* alleles, i.e., N-glycosylation, phosphorylation, tyrosine sulfation, N-myristoylation, and leucine zipper. These sites were present in variable numbers in all the *Pi54* alleles except for tyrosine kinase phosphorylation site and leucine zipper. The Zn-finger domain protein is reported responsive to wounding, stress hormones, cold, salt, submergence, heavy metals and desiccation (Vij and Tyagi, 2008). The presence of motif sites in the translated sequence indicate its role in downstream signaling of defense response genes (callose, laccase, PAL, and peroxidase), transcription factors (NAC6, Dof Zinc finger, MAD box, bZIP, and WRKY) that fortify cell wall/plasmodesmata leading to hypersensitive response, and affecting resistance reaction (Gupta et al., 2011).

In the study, fifty new haplotypes were identified from the nucleotide polymorphism of the *Pi54* alleles. Interestingly, we identified one haplotype which is resistant specific (H_3). Small number of haplotypes was detected previously within the gene pool of cultivated (*H. vulgare*) barley. In *Hordeum* species, the total number of haplotypes identified (46) in *H. spontaneum* almost double from those detected in *H. vulgare* (Russell et al., 2004). Similarly, 16 haplotypes were identified from nucleotide polymorphism of fiftyone *Pi-ta* alleles (Huang et al., 2008). In another study, 53 *Pi-ta* haplotypes were identified from the nucleotide polymorphism of 229 rice accessions belonging to seven *Oryza* species. These findings highlighted the importance of analysis and utilization of haplotypes from landraces and related wild species for crop improvement (Lee et al., 2009).

Balancing and purifying selection have been observed for the evolution of *R*-genes. The value of Tajima's D was negative in the entire coding region (−1.03898) and LRR domain (−1.70363) indicating purifying selection in the CDS and LRR regions. Similar, values were also reported for *Pi-ta* alleles of *O. rufipogon* and *Pi9* alleles of five *Oryza* species (AA genome) (Lee et al., 2009; Liu et al., 2011). Our analyses implicate selection pressure high on both CDS and LRR domains of resistant lines, in contrast to the LRR domain of susceptible rice lines. Elucidation of the selection mechanism acting on the domains could help to design new crop improvement strategies for the future.

In the LRR region of the *Pi54* alleles of different groups, the value of k_a_/k_s_ > 1.0 implying positive directional selection might have favored amino acid substitution in the region. Similar results were also obtained in *Piz(t)* alleles of Indian landraces of diverse locations (Thakur et al., 2013b). In contrast, the LRR regions encoded by *Pi-km1* and *Pi-km2* blast resistance genes were highly conserved (Ashikawa et al., 2010). Importantly, LRRs have direct interacting roles with effector proteins (Young and Innes, 2006). Most isolated *R* -genes encode proteins possessing LRR domain, of which the majority also contains a NBS domain (Sharma et al., 2012). Higher level of polymorphism in the LRR region as obtained in case of *Pi54* gene is thought to be involved in the recognition of effector proteins, and consequently the evolutionary pressure on the host by virulent *M. oryzae* races results in high variability in LRR domain. The LRR regions of many *Arabidopsis R* genes have k_a_/k_s_ ratios >1, suggesting that these *R* genes have evolved under positive selection pressure (Bergelson et al., 2001). Two basic strategies have evolved for an R protein to recognize a pathogen effector (which is also called avirulence (Avr) factor): direct physical interaction and indirect interaction *via*. association with other host proteins targeted by the Avr factor (Xiao et al., 2008). It has also been reported that variation for disease resistance is maintained by frequency-dependent selection, even though there is a fitness cost associated with the maintenance of *R* genes in the absence of their matching *Avr* (Stahl et al., 1999). Flax *L* genes and their matching *Avr* genes in flax rust undergo strong diversifying selection, suggesting direct interaction (Dodds et al., 2006). However, in case of *A. thaliana* indirect interaction between *RPM1* and *AvrB*, in which the RIN4 protein acts as a target for binding to *AvrB*, and the AvrB-induced phosphorylation of RIN4 then activates *RPM1* has been reported as balancing selection (Mackey et al., 2002).

Development of allele specific functional markers holds the key for marker assisted selection. These functional markers based on genic *Pi54* InDels can be applicable in MAS for blast resistance breeding programme. Absence of diagnostic bands in resistant/susceptible cultivars might be because of genetic recombination during meiotic cell cycle. However, the present study extends the repertoire of functional markers toward screening of genotypes. Similar functional InDel-based marker has been reported to be developed for blast resistance gene *Pikm* (Costanzo and Jia, 2010). The sequence of nine blast resistance genes was used for the development of functional markers based on InDels (Hayashi et al., 2006). However, allele specific markers are not known for blast resistance gene *Pi54*, hence the markers developed in this study would be of great significance to the breeders. Increasing efforts to clone more resistance genes worldwide will accelerate the development of more dominant resistance gene based markers for molecular breeding, thereby accelerating introduction of durable, broad-spectrum blast resistant genes into widely adapted high yielding rice cultivars.

From the above discussion, we conclude that the nucleotide variation was high in the LRR domain of all the *Pi54* alleles cloned and characterized in this study. In disease resistant alleles, selection pressure was high in LRR domain and CDS region whereas in susceptible counterpart selection pressure exerted only in the LRR domain. It was also evident that LRR domain of *Pi54* alleles was diversified because of high selection pressure. The co-dominant and dominant functional markers developed in the present study can be used in marker-assisted breeding programs aimed at improvement of blast resistance in elite rice cultivars. The diversity information based on genetic structure is an extremely important pre-breeding material in selecting parents for intra- and inter- group crosses to broaden the genetic base of modern rice cultivars. This study helps understand the extent of variability present in the landraces and cultivated varieties of rice that can be employed in future for selection of better alleles and their utilization in resistant breeding programmes.

## Author contributions

Conceived and designed the experiments: TRS. Performed the experiments and analyzed data: ST. Contributed materials/analysis tools: RR, MV, SP, DC, AD, NS, AS, UD, and PS. Wrote the paper: TRS, ST, and AD.

### Conflict of interest statement

The authors declare that the research was conducted in the absence of any commercial or financial relationships that could be construed as a potential conflict of interest.

## Supplementary material

Figure S1 **Haplotype network based on 187 potential SNPs of the *Pi54* resistant alleles**. Each group of haplotypes is shown as a solid circle, and five major haplotypes are marked in larger circles. Each branch represents a single mutational step. Branches with small solid circles indicate that there is more than a single mutational step between haplotypes. Different sizes of circles represent the different numbers of each haplotype.

Figure S2 **Haplotype network based on 97 potential SNPs of the *Pi54* susceptible alleles**. Each group of haplotypes is shown as a solid circle, and four major haplotypes are marked in larger circles. Each branch represents a single mutational step. Branches with small solid circles indicate that there is more than a single mutational step between haplotypes. Different sizes of circles represent the different numbers of each haplotype.

Figure S3 **Analysis of ZnF domain of 61 Pi54 proteins**. Arrows indicate that the ZnF doamain was not predicted inspite of the sequence of the ZnF in the Pi54 proteins. Single amino acid substitution in two Pi54 proteins has been encircled.

Figure S4 **Phylogenetic tree of LRR domain of 64 Pi54 protein**. Neighbor-Joining method was used for the construction of tree. Definite clustering was not obtained in all the tees.
