# Supplementary material for: Extensive sequence variation in rice blast resistance gene Pi54 makes it broad spectrum in nature
Source: Front Plant Sci. 2015 May 21;6:345. doi: 10.3389/fpls.2015.00345 (PMC4440361; doi:10.3389/fpls.2015.00345)
Supplement: Figure S1 — Haplotype network based on 187 potential SNPs of the Pi54 resistant alleles. Each group of haplotypes is shown as a solid circle, and five major haplotypes are marked in larger circles. Each branch represents a single mutational step. Branches with small solid circles indicate that there is more than a single mutational step between haplotypes. Different sizes of circles represent the different numbers of each haplotype. [file Presentation1.PPT]

## Slide 1
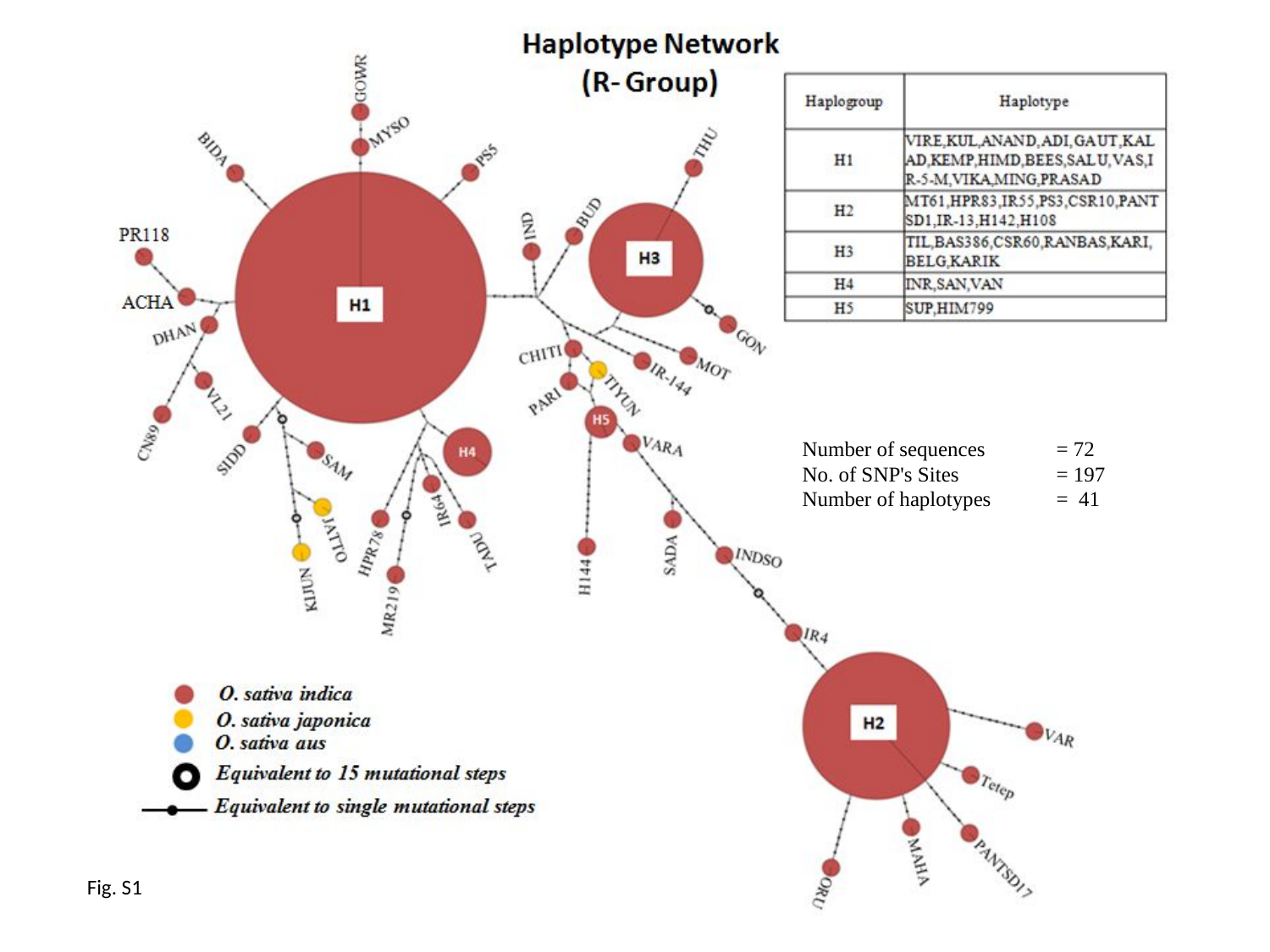

Number of sequences	= 72
No. of SNP's Sites	= 197
Number of haplotypes 	= 41
Fig. S1

## Slide 2
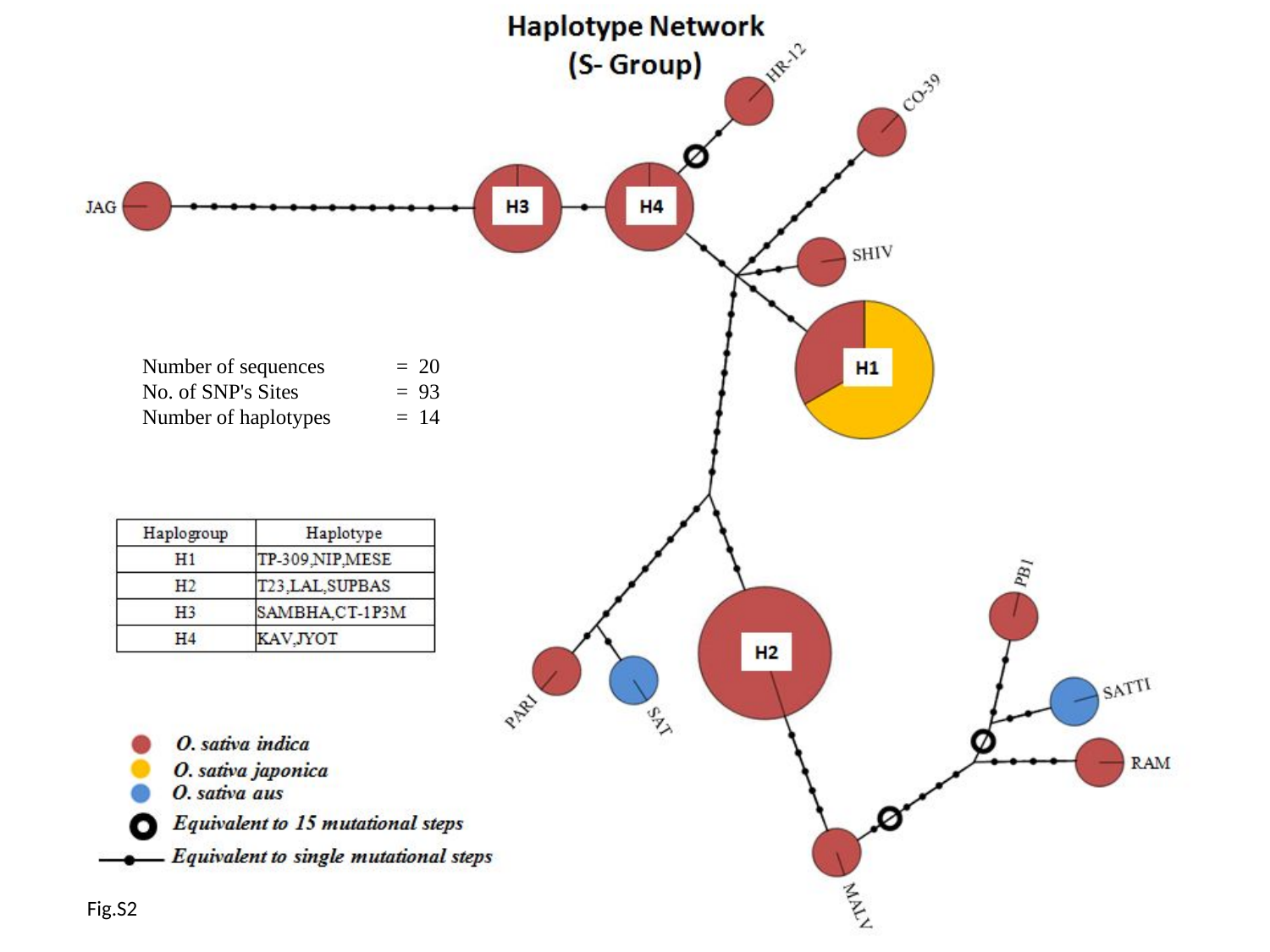

Number of sequences	= 20
No. of SNP's Sites	= 93
Number of haplotypes 	= 14
Fig.S2

## Slide 3
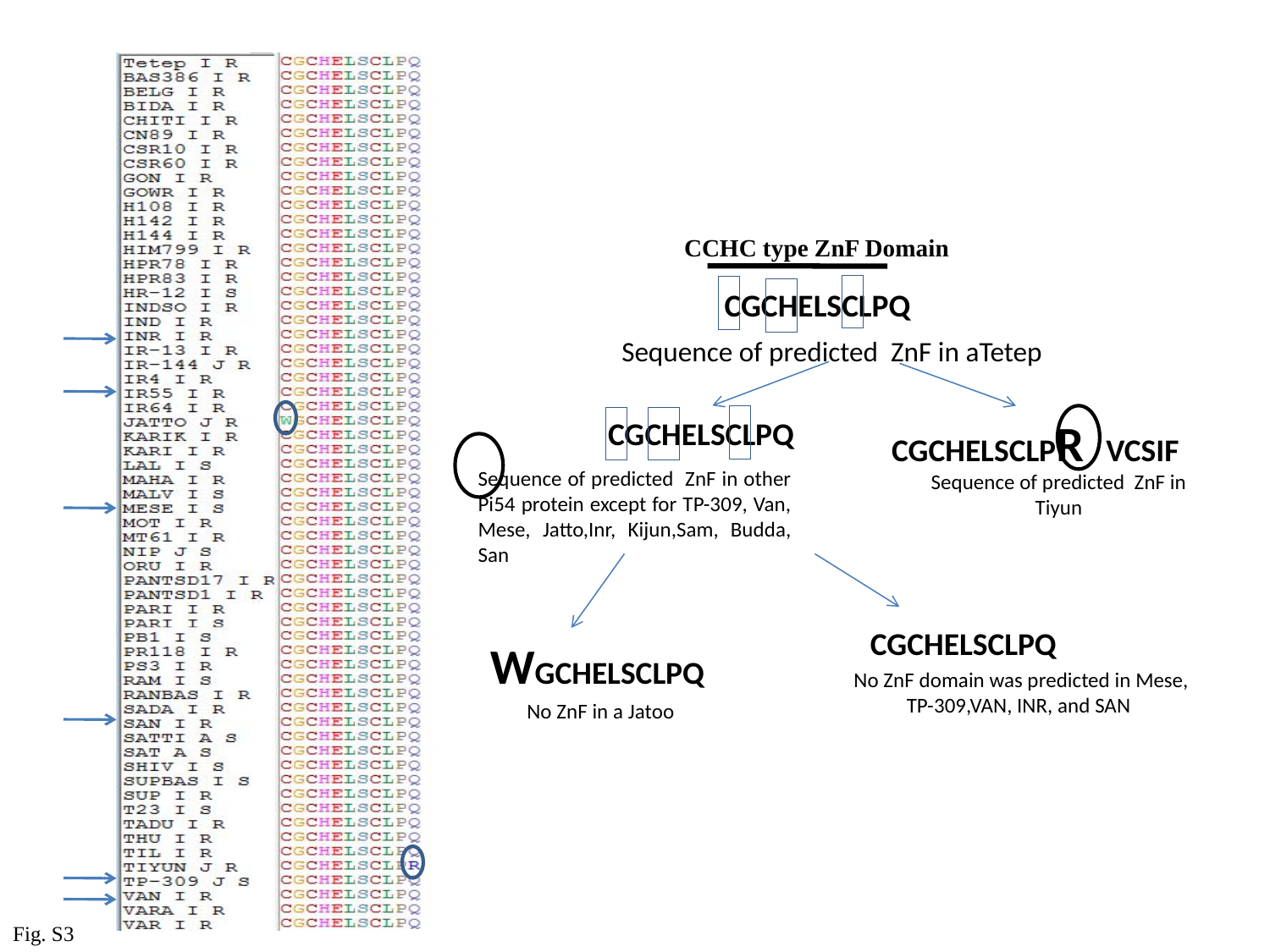

CCHC type ZnF Domain
CGCHELSCLPQ
Sequence of predicted ZnF in aTetep
CGCHELSCLPQ
Sequence of predicted ZnF in other Pi54 protein except for TP-309, Van, Mese, Jatto,Inr, Kijun,Sam, Budda, San
WGCHELSCLPQ
No ZnF in a Jatoo
CGCHELSCLPR VCSIF
Sequence of predicted ZnF in Tiyun
CGCHELSCLPQ
No ZnF domain was predicted in Mese, TP-309,VAN, INR, and SAN
Fig. S3

## Slide 4
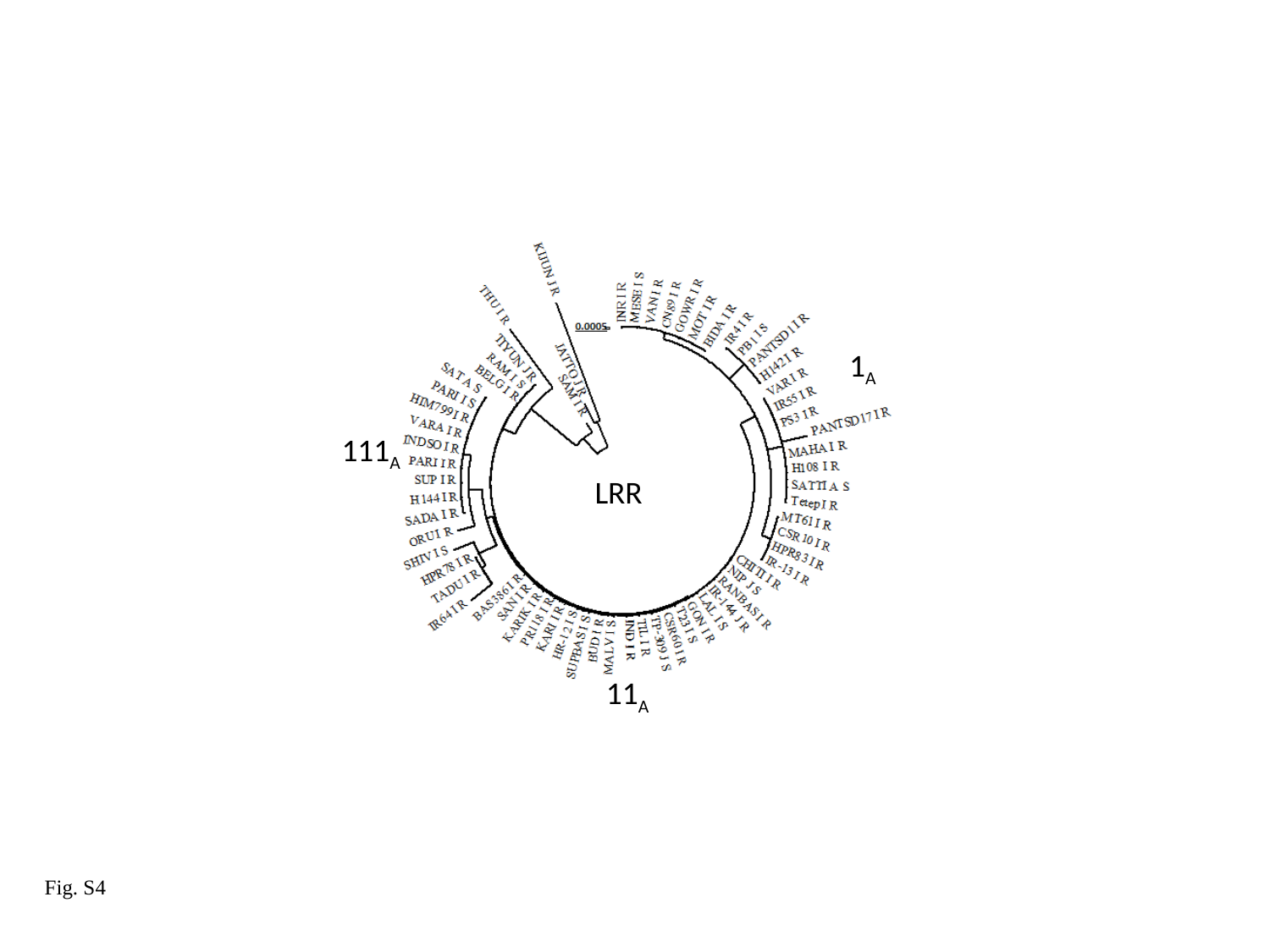

1A
111A
LRR
11A
Fig. S4
