# Supplementary material for: Extensive sequence variation in rice blast resistance gene Pi54 makes it broad spectrum in nature
Source: Front Plant Sci. 2015 May 21;6:345. doi: 10.3389/fpls.2015.00345 (PMC4440361; doi:10.3389/fpls.2015.00345)
Supplement: Supplementary file 2 [file Table1.DOC]

| **Table S1** Origin and distribution of 92 *Pi54* alleles along with phenotype and size of amplified allele | | | | | | | | | |
| --- | --- | --- | --- | --- | --- | --- | --- | --- | --- |
| **Sample Name** | **Sample ID** | **Accession NO.** | **Species** | **Group** | **Source** | **State/origin** | **Seq. Length** | **Reaction Type** | **Phenotype** |
| Acharmati | ACHA | HE586221 | *O. sativa indica* | Landrace | Odisha | Odisha | 1935 | 1 | R |
| Aditya | ADI | HE586233 | *O. sativa indica* | Cultivar | DRR, Hyderabad | Andhra Pradesh | 2024 | 3 | R |
| Annada | ANAND | HE586238 | *O. sativa indica* | Cultivar | Cuttack | Odisha | 2011 | 1 | R |
| Basmati 386 | BAS 386 | HE586211 | *O. sativa indica* | Cultivar | Ludhiana | Punjab | 2240 | 2 | R |
| Beesginsali | BEES | HE586161 | *O. sativa indica* | Landrace | Dharwad | Karnataka | 1851 | 1 | R |
| Belgaum basmati | BELG | HE586171 | *O. sativa indica* | Landrace | Belgaum | Karnataka | 1690 | 0 | R |
| Bidarlocal-2 | BIDA | HE586165 | *O. sativa indica* | Landrace | Bidar | Karnataka | 1851 | 2 | R |
| Budda | BUD | HE586186 | *O. sativa indica* | Landrace | Haveri | Karnataka | 1730 | 2 | R |
| Chiti zhini | CHITI | HE586169 | *O. sativa indica* | Landrace | Kangra | Himachal Pradesh | 1695 | 3 | R |
| CN-1789 | CN89 | HE586205 | *O. sativa indica* | Landrace | Chinsurah | West Bengal | 1974 | 1 | R |
| CO-39 | CO-39 | HE586193 | *O. sativa indica* | Cultivar | TNAU, Coimbatore | Tamil Nadu | 1997 | 4 | S |
| CSR 10 | CSR10 | HE586204 | *O. sativa indica* | Cultivar | Karnal | Haryana | 1793 | 1 | R |
| CSR-60 | CSR 60 | HE586208 | *O. sativa indica* | Cultivar | Karnal | Haryana | 1804 | 1 | R |
| CT-10006-7-2M-5-1P3M | CT-1P3M | HE586244 | *O. sativa indica* | Breeding line | Columbia | Columbia | 2041 | 5 | S |
| Dhanaprasad | DHAN | HE586223 | *O. sativa indica* | Landrace | Maharashtra | Maharashtra | 1941 | 1 | R |
| Gautam | GAUT | HE586247 | *O. sativa indica* | Cultivar | PUSA | Bihar | 2071 | 1 | R |
| Gonrra bhog | GON | HE586195 | *O. sativa indica* | Landrace | NA | NA | 1810 | 1 | R |
| Gowrisanna | GOWR | - | *O. sativa indica* | Landrace | Uttarakannada | Karnataka | 1851 | 2 | R |
| Himalya 799 | HIM 799 | HE586209 | *O. sativa indica* | Cultivar | Malan | Himachal Pradesh | 1822 | 2 | R |
| Himdhan | HIM D | HE586220 | *O. sativa indica* | Cultivar | Malan | Himachal Pradesh | 2076 | 3 | R |
| HLR-108 | H108 | HE586177 | *O. sativa indica* | Landrace | Kangra | Himachal Pradesh | 1657 | 3 | R |
| HLR-142 | H142 | HE586176 | *O. sativa indica* | Landrace | Shimla | Himachal Pradesh | 1642 | 3 | R |
| HPR 2083 | HPR 83 | HE586240 | *O. sativa indica* | Cultivar | Malan | Himachal Pradesh | 1650 | 1 | R |
| HPR-2178 | HPR 78 | HE586167 | *O. sativa indica* | Breeding line | Malan | Himachal Pradesh | 1663 | 3 | R |
| HR-12 | HR-12 | HE586159 | *O. sativa indica* | Cultivar | Hyderabad | Andhra Pradesh | 1647 | 5 | S |
| Indira sona | IND SO | HE586213 | *O. sativa indica* | Hybrid | Raipur | Chhattisgarh | 1797 | 1 | R |
| Indrayani | IND | HE586180 | *O. sativa indica* | Cultivar | Belgaum | Karnataka | 1802 | 1 | R |
| INRC 779 | INR | HE586181 | *O. sativa indica* | Landrace | Hazaribagh | Jharkhand | 2022 | 3 | R |
| IR 64 | IR 64 | HE586256 | *O. sativa indica* | Cultivar | IRRI | Philippines | 2018 | 2 | R |
| IRAT-144 | IR-144 | HE586255 | *O. sativa japonica* | Cultivar | Hazaribagh | Ivory Coast | 1628 | 3 | R |
| IRBB 55 | IR55 | HE586203 | *O. sativa indica* | Cultivar | IRRI | Philippines | 2236 | 1 | R |
| IRBB-13 | IR-13 | HE586246 | *O. sativa indica* | Cultivar | IRRI | Philippines | 1781 | 3 | R |
| IRBB-4 | IR 4 | HE586234 | *O. sativa indica* | Cultivar | IRRI | Philippines | 2173 | 1 | R |
| IRBLB-BIRBLB-5-M | IR-5-M | HE586226 | *O. sativa indica* | Cultivar | IRRI | Philippines | 2056 | 3 | R |
| Jagannath | JAG | HE586202 | *O. sativa indica* | Cultivar | Odisha | Odisha | 2073 | 4 | S |
| Jatto | JATTO | - | *O. sativa japonica* | Landrace | Kullu | Himachal Pradesh | 1687 | 3 | R |
| Jyoti | JYOT | HE586241 | *O. sativa indica* | Cultivar | Kerala | Kerala | 2121 | 4 | S |
| Kala Dhan | KALA D | HE586154 | *O. sativa indica* | Landrace | Kangra | Himachal Pradesh | 1850 | 3 | R |
| Kari kantiga | KARI K | HE586174 | *O. sativa indica* | Landrace | Uttarakannada | Karnataka | 1687 | 1 | R |
| Kariya | KARI | HE586170 | *O. sativa indica* | Landrace | Uttarakannada | karnataka | 1672 | 3 | R |
| Kavali kannu | KAV | HE586187 | *O. sativa indica* | Landrace | Dakshin Kannada | Karnataka | 2021 | 4 | S |
| Kempusali | KEMP | HE586158 | *O. sativa indica* | Landrace | Belgaum | Karnataka | 1850 | 2 | R |
| Kijun | KIJUN | - | *O. sativa japonica* | Landrace | Chamba | Himachal Pradesh | 1684 | 3 | R |
| Kulanji pille | KUL | HE586190 | *O. sativa indica* | Landrace | Dakshin Kannada | Karnataka | 1817 | 3 | R |
| Lalnakanda | LAL | HE586217 | *O. sativa indica* | Landrace | Ludhiana | Punjab | 1815 | 4 | S |
| HLR-144 | H144 | HE586183 | *O. sativa indica* | Landrace | Shimla | Himachal Pradesh | 1763 | 3 | R |
| Mahamaya | MAHA | HE586253 | *O. sativa indica* | Cultivar | Raipur | Chattisgarh | 1792 | 1 | R |
| Malviya dhan | MALV | HE586251 | *O. sativa indica* | Cultivar | Varanasi | Uttarpradesh | 1823 | 4 | S |
| Mesebatta | MESE | HE586173 | *O. sativa indica* | Landrace | Uttarakannada | Karnataka | 1878 | 5 | S |
| Mingola | MING | HE586192 | *O. sativa indica* | Landrace | Uttarakannada | Karnataka | 2176 | 2 | R |
| Mote bangarkaddi | MOT | HE586184 | *O. sativa indica* | Landrace | Uttarakannada | Karnataka | 1937 | 3 | R |
| MR 219 | MR 219 | HE586216 | *O. sativa indica* | Cultivar | NA | Malaysia | 1926 | 2 | R |
| MTU-1061 | MT61 | HE586196 | *O. sativa indica* | Cultivar | Maruteru | Andhra Pradesh | 2151 | 1 | R |
| Mysore mallige | MYSO | HE586162 | *O. sativa indica* | Landrace | Mysore | Karnataka | 1845 | 2 | R |
| Nipponbare | NIP | - | *O. sativa japonica* | Cultivar | Japan | Japan | 1669 | 5 | S |
| Orugallu | ORU | HE586163 | *O. sativa indica* | Cultivar | Haveri | Karnataka | 1466 | 2 | R |
| Pant sankar dhan 1 | PANT SD 1 | HE586243 | *O. sativa indica* | Cultivar | Pantnagar | Uttarakhand | 1782 | 1 | R |
| Pant sugandh dhan 17 | PANT SD 17 | HE586215 | *O. sativa indica* | Cultivar | Pantnagar | Uttarakhand | 1789 | 1 | R |
| Parijat | PARI | HE586225 | *O. sativa indica* | Cultivar | Odisha | Odisha | 1820 | 4 | S |
| Parimala kalvi | PARI | HE586172 | *O. sativa indica* | Landrace | Bidar | Karnataka | 1696 | 3 | R |
| *Pi54* (Tetep) | Tetep | AY914077 | *O. sativa indica* | Cultivar | Indonesia | Indonesia | 1459 | 0 | R |
| PR 118 | PR 118 | HE586252 | *O. sativa indica* | Cultivar | Ludhiana | Punjab | 1829 | 0 | R |
| Prasad | PRASAD | HE586232 | *O. sativa indica* | Cultivar | Pantnagar | Uttarakhand | 2078 | 1 | R |
| Pusa basmati 1 | PB 1 | HE586254 | *O. sativa indica* | Cultivar | IARI | Delhi | 1787 | 4 | S |
| Pusa Sugandh 3 | PS 3 | HE586249 | *O. sativa indica* | Cultivar | IARI | Delhi | 1779 | 2 | R |
| Pusa sugandh 5 | PS 5 | HE586257 | *O. sativa indica* | Cultivar | IARI | Delhi | 1899 | 3 | R |
| Ram Jawain 100 | RAM | HE586164 | *O. sativa indica* | Cultivar | Kangra | Himachal Pradesh | 1466 | 4 | S |
| Ranbir basmati | RAN BAS | HE586212 | *O. sativa indica* | Cultivar | Jammu | J&K | 1816 | 0 | R |
| Sadabahar | SADA | HE586236 | *O. sativa indica* | Cultivar | Hazaribagh | Jharkhand | 1771 | 2 | R |
| Salumpikit | SALU | HE586175 | *O. sativa indica* | Cultivar | Hazaribagh | Jharkhand | 1959 | 1 | R |
| Samba mahsuri | SAMBHA | HE586229 | *O. sativa indica* | Cultivar | Hyderabad | Andhra Pradesh | 1983 | 4 | S |
| Samleshwari | SAM | HE586200 | *O. sativa indica* | Cultivar | Raipur | Chattisgarh | 1956 | 0 | R |
| Sanna mullare | SAN | HE586188 | *O. sativa indica* | Cultivar | Uttarakannada | Karnataka | 1881 | 3 | R |
| Sathia -2 | SAT | HE586182 | *O. sativa aus* | Landrace | Hazaribagh | Jharkhand | 1828 | 4 | S |
| Satti | SATTI | HE586206 | *O. sativa aus* | Landrace | NA | Gujarat | 1781 | 5 | S |
| Shiva | SHIV | HE586210 | *O. sativa indica* | Cultivar | Hyderabad | Andhra Pradesh | 2601 | 4 | S |
| Siddasala | SIDD | HE586155 | *O. sativa indica* | Landrace | Uttarakannada | Karnataka | 1850 | 1 | R |
| Superbasmati | SUP BAS | HE586224 | *O. sativa indica* | Cultivar | Ludhiana | Punjab | 1804 | 4 | S |
| Suphala | SUP | HE586219 | *O. sativa indica* | NA | NA | NA | 1813 | 0 | R |
| T23 | T23 | HE586250 | *O. sativa indica* | Cultivar | NA | NA | 1804 | 5 | S |
| Tadukan | TADU | HE586156 | *O. sativa indica* | Cultivar | Philippines | Philippines | 1646 | 1 | R |
| Taipei-309 | TP-309 | HE586157 | *O. sativa japonica* | Cultivar | Taipei | Taipei | 1687 | 5 | S |
| Thule atte | THU | HE586185 | *O. sativa indica* | Landrace | Hazaribagh | Sikkim | 1756 | 3 | R |
| Tilak chandan | TIL | HE586207 | *O. sativa indica* | Landrace | NA | Uttar Pradesh | 1793 | 0 | R |
| Tiyun | TIYUN | HE586168 | *O. sativa japonica* | Landrace | Chamba | Himachal Pradesh | 1674 | 3 | R |
| V L Dhan 21 | V L 21 | HE586214 | *O. sativa indica* | Cultivar | Malan | Uttarakhand | 1958 | 1 | R |
| Vanasurya | VAN | HE586189 | *O. sativa indica* | Landrace | Uttarakannada | Karnataka | 1956 | 2 | R |
| Varalu | VARA | HE586239 | *O. sativa indica* | Landrace | NA | Karnataka | 1809 | 1 | R |
| Varun dhan | VAR | HE586199 | *O. sativa indica* | Cultivar | Malan | Himachal Pradesh | 1754 | 3 | R |
| Vasanesanna batta | VAS | HE586191 | *O. sativa indica* | Landrace | Uttarakannada | Karnataka | 1991 | 2 | R |
| Vikash | VIKA | HE586235 | *O. sativa indica* | Cultivar | NA | NA | 2081 | 0 | R |
| Virendra | VIRE | HE586245 | *O. sativa indica* | Cultivar | Hazaribagh | Jharkhand | 2053 | 0 | R |

NA- not applicable

| **Table S2** Percentages of mutational change at the *Pi54* alleles obtained from blast resistance and susceptible accessions of *Oryza* species (Nmut ≥ 10) | | | | | | | |
| --- | --- | --- | --- | --- | --- | --- | --- |
| **Nucleotide position** | | **Mutational change** | **Transition/ Transversion (ti/tv)** | **Disease resistance phenotypes (n=72) % (n)** | **Disease susceptible phenotypes (n=20) % (n)** | | **Total % (n)** |
| 57 | | A→G | ti | 75.0 (54) | 80.0 (16) | | 76.1 (70) |
| 61 | | G→A | ti | 73.6 (53) | 80.0 (16) | | 75.0 (69) |
| 73 | | G→A | ti | 73.6 (53) | 80.0 (16) | | 75.0 (69) |
| 231 | | C→A | tv | 75.0 (54) | 80.0 (16) | | 76.1 (70) |
| 346 | | C→T | ti | 55.6 (40) | 55.0 (11) | | 55.4 (51) |
| 373 | | G→A | ti | 75.0 (54) | 80.0 (16) | | 76.1 (70) |
| 413 | | T→G | tv | 98.6 (71) | 100.0 (20) | | 98.9 (91) |
| 475 | | C→A | tv | 15.3 (11) | 20.0 (4) | | 16.3 (15) |
| 479 | | C→A/T(1R) | tv/ti | 66.7 (48) | 65.0 (13) | | 66.3 (61) |
| 504 | | T→C | ti | 12.5 (9) | 15.0 (3) | | 13.0 (12) |
| 629 | | T→C | ti | 79.2 (57) | 90.0 (18) | | 81.5 (75) |
| 669 | | T→C | ti | 79.2 (57) | 90.0 (18) | | 81.5 (75) |
| 755 | | C→T | ti | 37.5 (27) | 35.0 (7) | | 37.0 (34) |
| 803 | | T→G | tv | 79.2 (57) | 90.0 (18) | | 81.5 (75) |
| 813 | | C→T | ti | 15.3 (11) | 20.0 (4) | | 16.3 (15) |
| 852 | | C→T | ti | 79.2 (57) | 95.0 (19) | | 82.6 (76) |
| 883 | | G→A | ti | 12.5 (9) | 10.0 (2) | | 12.0 (11) |
| 970 | | C→G | tv | 12.5 (9) | 10.0 (2) | | 12.0 (11) |
| 972 | | T→A | tv | 79.2 (57) | 90.0 (18) | | 81.5 (75) |
| 979 | | T→A | tv | 80.6 (58) | 90.0 (18) | | 82.6 (76) |
| 984 | | T→G | tv | 79.2 (57) | 90.0 (18) | | 81.5 (75) |
| 1001 | | G→A | ti | 51.4 (37) | 60.0 (12) | | 53.3 (49) |
| 1008 | | G→A | ti | 80.6 (58) | 90.0 (18) | | 82.6 (76) |
| 1038 | | A→G | ti | 80.6 (58) | 90.0 (18) | | 82.6 (76) |
| 1039 | | C→T | ti | 79.2 (57) | 90.0 (18) | | 81.5 (75) |
| 1045 | | G→C | tv | 79.2 (57) | 90.0 (18) | | 81.5 (75) |
| 1064 | | C→G | tv | 79.2 (57) | 90.0 (18) | | 81.5 (75) |
| 1127 | | C→A | tv | 79.2 (57) | 85.0 (17) | | 80.4 (74) |
| 1140 | | T→C | ti | 77.8 (56) | 85.0 (17) | | 79.3 (73) |
| 1142 | | G→A | ti | 50.0 (36) | 55.0 (11) | | 51.1 (47) |
| 1143 | | T→C | ti | 79.2 (57) | 85.0 (17) | | 80.4 (74) |
| 1149 | | C→T | ti | 15.3 (11) | 10.0 (2) | | 14.1 (13) |
| 1164 | | A→G | ti | 76.4 (55) | 80.0 (16) | | 77.2 (71) |
| 1176 | | G→A | ti | 76.4 (55) | 80.0 (16) | | 77.2 (71) |
| 1179 | | T→G | tv | 76.4 (55) | 80.0 (16) | | 77.2 (71) |
| 1185 | | C→T | ti | 76.4 (55) | 80.0 (16) | | 77.2 (71) |
| 1201 | | A→G | ti | 50.0 (36) | 55.0 (11) | | 51.1 (47) |
| 1205 | | A→G | ti | 76.4 (55) | 80.0 (16) | | 77.2 (71) |
| 1254 | | G→A | ti | 15.3 (11) | 10.0 (2) | | 14.1 (13) |
| 1274 | | G→A | ti | 12.5 (9) | 20.0 (4) | | 14.1 (13) |
|  |  | | | | |  | |

| **Table S3** Summary of predicted gene statistics of *Pi54* allele in 92 accessions of *Oryza* species | |
| --- | --- |
| **Description** | **Gene statistics** |
| No significant gene prediction | 28 |
| No *poly A* prediction | 10 |
| Allele size range | 1459 - 2601 |
| One exon prediction | 40 |
| Two exon prediction | 21 |
| Three exon prediction | 3 |
| Smallest mRNA | 222 bp (Kijun_J_R) |
| Largest mRNA | 1461bp (IR4_I_R) |
| Smallest Protein | 73 aa (Kijun_J_R) |
| Largest Protein | 486 aa (IR4_I_R) |

| **Table S4** Percentages of amino acid substitutions at the *Pi54* gene observed in disease resistance and susceptible phenotypes of O*ryza* species (Nsites (s) ≥ 10) | | | |
| --- | --- | --- | --- |
| **Substitution position** | **Disease resistance phenotypes (n = 50) % (n)** | **Disease susceptible phenotypes (n = 14) % (n)** | **Total % (n)** |
| H57Y/Q(1) | 20.0 (10) | 28.6 (4) | 21.9 (14) |
| S66N/A(1) | 30.0 (15) | 35.7 (5) | 31.3 (20) |
| Y80H | 64.0 (32) | 71.4 (10) | 65.6 (42) |
| V81A | 28.0 (14) | 35.7 (5) | 29.7 (19) |
| M86I | 64.0 (32) | 71.4 (10) | 65.6 (42) |
| R88S | 64.0 (32) | 71.4 (10) | 65.6 (42) |
| M104L/I(1) | 44.0 (22) | 35.7 (5) | 42.2 (27) |
| A106S | 46.0 (23) | 35.7 (5) | 43.8 (28) |
| K109N/R(1) | 44.0 (22) | 35.7 (5) | 42.2 (27) |
| I116L | 44.0 (22) | 35.7 (5) | 42.2 (27) |
| A123V/K(1)/Q(1) | 44.0 (22) | 42.9 (6) | 43.8 (28) |
| G127R | 46.0 (23) | 50.0 (7) | 46.9 (30) |
| L131I | 44.0 (22) | 50.0 (7) | 45.3 (29) |
| S133C | 46.0 (23) | 50.0 (7) | 46.9 (30) |
| G135D | 44.0 (22) | 50.0 (7) | 45.3 (29) |
| Y155F | 46.0 (23) | 50.0 (7) | 46.9 (30) |
| S158T | 18.0 (9) | 14.3 (2) | 17.2 (11) |
| A187V | 18.0 (9) | 14.3 (2) | 17.2 (11) |
| M214L | 62.0 (31) | 85.7 (12) | 67.2 (43) |
| T272A/P(1) | 64.0 (32) | 85.7 (12) | 68.8 (44) |
| D322Y | 42.0 (21) | 57.1 (8) | 45.3 (29) |
| C323F | 20.0 (10) | 28.6 (4) | 21.9 (14) |
| M344L/V(1) | 70.0 (35) | 71.4 (10) | 70.3 (45) |

| **Table S5** Number of different motiffs identified in 64 Pi54 proteins obtained from 64 rice accessions | | | | | | | |
| --- | --- | --- | --- | --- | --- | --- | --- |
| **Protein ID** | **ASN_Glycosylation site** | **Tyrosine sulfation site** | **Protein kinase C phosphorylation site** | **Casein kinase II phosphorylation site** | **Tyrosine kinase phosphorylation site** | **N-myristoylation site** | **Leucine_Zipper Pattern** |
| CHITI_I_R | 5 | 0 | 4 | 13 | 1 | 5 | 246 - 267 LwmnnkmLysskegLrgveksL |
| CSR10_I_R | 5 | 0 | 4 | 13 | 1 | 5 | 0 |
| CSR60_I_R | 5 | 1 | 4 | 13 | 1 | 5 | 226 - 247 LwmnnkmLysskegLrgveksL |
| GON_I_R | 4 | 1 | 4 | 13 | 1 | 4 | 226 - 247 LwmnnkmLysskegLrgveksL |
| GOWR_I_R | 4 | 0 | 3 | 10 | 1 | 4 | 214 - 235 LwmnnkmLysskegLrgveksL |
| H108_I_R | 5 | 0 | 4 | 13 | 1 | 5 | 0 |
| H142_I_R | 5 | 0 | 4 | 13 | 1 | 5 | 0 |
| H144_I_R | 5 | 0 | 3 | 13 | 1 | 5 | 246 - 267 LwmnnkmLysskegLrgveksL |
| HIM799_I_R | 5 | 0 | 4 | 13 | 1 | 5 | 246 - 267 LwmnnkmLysskegLrgveksL |
| HPR78_I_R | 4 | 0 | 1 | 9 | 1 | 4 | 172 - 193 LwmnnkmLysskegLrgveksL |
| HPR83_I_R | 4 | 0 | 4 | 13 | 1 | 4 | 0 |
| HR-12_I_S | 2 | 0 | 2 | 8 | 1 | 2 | 0 |
| IND_I_R | 5 | 0 | 4 | 13 | 1 | 8 | 246 - 267 LwmnnkmLysskegLrgveksL |
| INDSO_I_R | 5 | 0 | 4 | 13 | 1 | 5 | 246 - 267 LwmnnkmLysskegLrgveksL |
| INR_I_R | 2 | 0 | 4 | 8 | 1 | 2 | 114 - 135 LwmnnkmLysskegLrgveksL |
| IR-13_I_R | 5 | 0 | 4 | 13 | 1 | 5 | 0 |
| IR-144_J_R | 4 | 0 | 4 | 13 | 1 | 4 | 200 - 221 LwmnnkmLysskegLrgveksL |
| IR4_I_R | 7 | 0 | 4 | 16 | 1 | 6 | 0 |
| IR55_I_R | 6 | 0 | 4 | 15 | 1 | 5 | 0 |
| IR64_I_R | 2 | 0 | 2 | 8 | 1 | 2 | 0 |
| JATTO_J_R | 1 | 0 | 4 | 6 | 1 | 1 | 0 |
| KARI_I_R | 5 | 1 | 4 | 13 | 1 | 5 | 226 - 247 LwmnnkmLysskegLrgveksL |
| KARIK_I_R | 5 | 1 | 4 | 13 | 1 | 5 | 226 - 247 LwmnnkmLysskegLrgveksL |
| KIJUN_J_R | 1 | 0 | 0 | 3 | 1 | 0 | 0 |
| LAL_I_S | 5 | 1 | 4 | 13 | 1 | 5 | 226 - 247 LwmnnkmLysskegLrgveksL |
| MAHA_I_R | 5 | 0 | 4 | 13 | 1 | 5 | 0 |
| MALV_I_S | 5 | 0 | 3 | 11 | 0 | 4 | 163 - 184 LwmnnkmLysskegLrgveksL |
| MESE_I_S | 2 | 0 | 4 | 8 | 1 | 2 | 114 - 135 LwmnnkmLysskegLrgveksL |
| MOT_I_R | 2 | 0 | 4 | 8 | 1 | 2 | 114 - 135 LwmnnkmLysskegLrgveksL |
| MT61_I_R | 4 | 0 | 4 | 13 | 1 | 4 | 0 |
| NIP_J_S | 2 | 0 | 4 | 9 | 1 | 2 | 130 - 151 LwmnnkmLysskegLrgveksL |
| ORU_I_R | 4 | 0 | 4 | 13 | 1 | 4 | 0 |
| PANTSD1_I_R | 5 | 0 | 4 | 13 | 1 | 5 | 0 |
| PANTSD17_I_R | 5 | 0 | 3 | 11 | 1 | 4 | 0 |
| PARI_I_R | 5 | 0 | 4 | 13 | 1 | 5 | 246 - 267 LwmnnkmLysskegLrgveksL |
| PARI_I_S | 5 | 0 | 4 | 13 | 1 | 5 | 246 - 267 LwmnnkmLysskegLrgveksL |
| PB1_I_S | 5 | 0 | 4 | 13 | 1 | 5 | 0 |
| PR118_I_R | 2 | 0 | 2 | 7 | 1 | 2 | 0 |
| PS3_I_R | 5 | 0 | 4 | 13 | 1 | 5 | 0 |
| RAM_I_S | 4 | 0 | 4 | 12 | 1 | 5 | 200 - 221 LwmnnkmLysskegLrgveksL |
| RANBAS_I_R | 5 | 1 | 4 | 13 | 1 | 5 | 226 - 247 LwmnnkmLysskegLrgveksL |
| SADA_I_R | 5 | 0 | 4 | 13 | 1 | 5 | 246 - 267 LwmnnkmLysskegLrgveksL |
| SAM_I_R | 1 | 0 | 1 | 4 | 1 | 0 | 0 |
| SAN_I_R | 2 | 0 | 4 | 8 | 1 | 2 | 114 - 135 LwmnnkmLysskegLrgveksL |
| SAT_A_S | 5 | 0 | 3 | 13 | 1 | 5 | 246 - 267 LwmnnkmLysskegLrgveksL |
| SATTI_A_S | 5 | 0 | 4 | 13 | 1 | 5 | 0 |
| SHIV_I_S | 3 | 0 | 2 | 11 | 1 | 3 | 0 |
| SUP_I_R | 5 | 0 | 4 | 13 | 1 | 5 | 246 - 267 LwmnnkmLysskegLrgveksL |
| SUPBAS_I_S | 5 | 1 | 4 | 13 | 1 | 5 | 226 - 247 LwmnnkmLysskegLrgveksL |
| T23_I_S | 5 | 1 | 4 | 13 | 1 | 5 | 226 - 247 LwmnnkmLysskegLrgveksL |
| TADU_I_R | 2 | 0 | 2 | 8 | 1 | 2 | 0 |
| Tetep_I_R | 4 | 0 | 4 | 13 | 1 | 4 | 0 |
| THU_I_R | 5 | 1 | 3 | 11 | 1 | 4 | 226 - 247 LwmnnkmLysskegLrgveksL |
| TIL_I_R | 5 | 1 | 4 | 13 | 1 | 5 | 226 - 247 LwmnnkmLysskegLrgveksL |
| TIYUN_J_R | 0 | 0 | 0 | 0 | 1 | 4 | 246 - 267 LwmnnkmLysskegLrgveksL |
| TP-309_J_S | 2 | 0 | 4 | 8 | 1 | 2 | 114 - 135 LwmnnkmLysskegLrgveksL |
| VAN_I_R | 2 | 0 | 4 | 8 | 1 | 2 | 114 - 135 LwmnnkmLysskegLrgveksL |
| VAR_I_R | 4 | 0 | 4 | 11 | 1 | 3 | 0 |
| VARA_I_R | 5 | 0 | 4 | 13 | 1 | 5 | 246 - 267 LwmnnkmLysskegLrgveksL |
| BAS386_I_R | 7 | 1 | 4 | 15 | 1 | 6 | 305 - 326 LwmnnkmLysskegLrgveksL |
| BELG_I_R | 0 | 1 | 4 | 12 | 1 | 5 | 239 - 247 LwmnnkmLysskegLrgveksL |
| BIDA_I_R | 2 | 0 | 2 | 7 | 1 | 2 | 0 |
| Budda_1_R | 2 | 0 | 0 | 3 | 0 | 0 | 0 |
| CN89_I_R | 2 | 0 | 4 | 7 | 1 | 2 | 114 - 135 LwmnnkmLysskegLrgveksL |
